# QTL mapping of the production of wine aroma compounds by yeast

**DOI:** 10.1186/1471-2164-13-573

**Published:** 2012-10-30

**Authors:** Damien Steyer, Chloe Ambroset, Christian Brion, Patricia Claudel, Pierre Delobel, Isabelle Sanchez, Claude Erny, Bruno Blondin, Francis Karst, Jean-Luc Legras

**Affiliations:** 1 INRA, UMR1131, Colmar, F-68021, France; 2 Université de Strasbourg, UMR1131, Strasbourg, F-68021, France; 3 Twistaroma, Colmar, F-68021, France; 4 INRA, UMR1083, Montpellier, F-34060, France; 5 Montpellier SupAgro, UMR1083, Montpellier, F-34060, France; 6 Université Montpellier 1, UMR1083, Montpellier, F-34060, France; 7 Université de Haute Alsace, EA3991 Laboratoire Vigne Biotechnologies et Environnement, Colmar, F-68021, France

**Keywords:** Saccharomyces cerevisiae, QTL mapping, Wine aroma, Citronellol, Rose oxide, Nerolidol, Farnesene, Ethyl esters, 2-phenyl ethanol, *PDR8*, *ABZ1*, *PLB2*, *QDR2*

## Abstract

**Background:**

Wine aroma results from the combination of numerous volatile compounds, some produced by yeast and others produced in the grapes and further metabolized by yeast. However, little is known about the consequences of the genetic variation of yeast on the production of these volatile metabolites, or on the metabolic pathways involved in the metabolism of grape compounds. As a tool to decipher how wine aroma develops, we analyzed, under two experimental conditions, the production of 44 compounds by a population of 30 segregants from a cross between a laboratory strain and an industrial strain genotyped at high density.

**Results:**

We detected eight genomic regions explaining the diversity concerning 15 compounds, some produced *de novo* by yeast, such as nerolidol, ethyl esters and phenyl ethanol, and others derived from grape compounds such as citronellol, and cis-rose oxide. In three of these eight regions, we identified genes involved in the phenotype. Hemizygote comparison allowed the attribution of differences in the production of nerolidol and 2-phenyl ethanol to the *PDR8* and *ABZ1* genes, respectively. Deletion of a *PLB2* gene confirmed its involvement in the production of ethyl esters. A comparison of allelic variants of *PDR8* and *ABZ1* in a set of available sequences revealed that both genes present a higher than expected number of non-synonymous mutations indicating possible balancing selection.

**Conclusions:**

This study illustrates the value of QTL analysis for the analysis of metabolic traits, and in particular the production of wine aromas. It also identifies the particular role of the *PDR8* gene in the production of farnesyldiphosphate derivatives, of *ABZ1* in the production of numerous compounds and of *PLB2* in ethyl ester synthesis. This work also provides a basis for elucidating the metabolism of various grape compounds, such as citronellol and cis-rose oxide.

## Background

The wide diversity that can be observed among individuals of the same species is one of the most remarkable aspects of life. Deciphering the mechanisms explaining this phenotypic variety is among the major aims of evolutionists and geneticists. Quantitative genetics has been applied to untangle these issues, and over the last 30 years numerous studies have illustrated the power of these genetic approaches, and in particular quantitative trait locus (QTL) mapping, with the characterization of many genomic regions linked to or containing genes responsible for quantitative variations in a phenotype. These approaches have been extensively used in plant and cattle breeding programs; they have contributed to the understanding of resistance to several diseases [1] and also led to a significant improvement in crop yields and cattle breeding. Surprisingly quantitative genetic approaches have been applied only recently to budding yeast, initially to elucidate various complex mechanisms, including sporulation efficiency [2], thermotolerance [3,4], and drug resistance [5]. Even more recently, this quantitative approach has been used to decipher complex traits [6,7] at high resolution [8,9]. It has also been applied successfully to the analysis of variations in gene expression [10,11]. The QTL approach is now being used to study features important for the beverages industry, for example wine fermentation [12,13], sake technological traits [14] and ethanol tolerance for ethanol production [15]. All these studies have implicated defective alleles, of for example *AMN1*[10], *ASP1*[16] or *ABZ1*[11], in the diversity of the phenotypes of segregants. Strain By4741 possesses a defective allele of *AMN1* which leads to faster daughter cell separation; wine strain SB possesses a defective allele of the *ASP1* gene involved in asparagine catabolism; and S288C possesses a defective allele of *ABZ1* that codes for an enzyme which catalyzes the synthesis of 4-amino-4- deoxychorismate from chorismate, a step in the synthesis of paraminobenzoic acid. This defective allele of *ABZ1* modulates the fermentation rate by controlling nitrogen utilization [11].

Wine aroma is complex and results from the blending of numerous compounds synthesized by vines, some of which are transformed by yeast, together with compounds directly produced by yeast as a result of its primary metabolism [17,18]. The metabolic pathways leading to the synthesis of these yeast volatile compounds are numerous and incompletely described. The roles of some of the key genes, such as *ATF1* for acetates and *EEB1* for ethyl esters, has been demonstrated [19,20]. Nevertheless, little is known about the factors explaining large strain-to-strain differences in the production of volatile compounds [21-23]. Holistic approaches [24] have given new insights into the roles of various key genes in the diversity of production of some volatile compounds. Further work from the same group highlighted how a few key players, such as transcription factors, may explain some of the differences between strains [25].

To analyze the differences in the production of wine aroma compounds linked to yeast strain diversity, we used QTL analysis with a population of 30 segregants arising from a cross between the laboratory strain S288C and 59A, a spore isolated from the industrial wine strain EC1118. This population of segregants has been genotyped with Affymetrix YGS98 microarrays to obtain a high density genetic map and was used for the first quantitative analysis of transcriptome variations during enological fermentation [11]. We tested this population of segregants for the production of aromatic compounds in two different experimental conditions: synthetic musts mimicking white and red wine fermentations. These analyses enabled us to detect the involvement of eight genomic regions in the production of various volatile compounds explaining 39% to 72% of the diversity. As examples, we characterized the role of two genes by hemizygote analysis and identified another candidate gene by analysis of the phenotype of a deleted mutant. Our findings provide new insights into the genetic architecture underlying the production of wine aroma by yeast.

## Results

Each of the 30 segregants was tested in two experimental designs. In the first design (experiment A), white wine fermentation was simulated by fermentation at 20°C in medium with a low lipid content, whereas in the second design, mimicking red wine fermentation [11], the fermentations were run at 28°C in medium with a high lipid content (experiment B).

**Figure 1:**
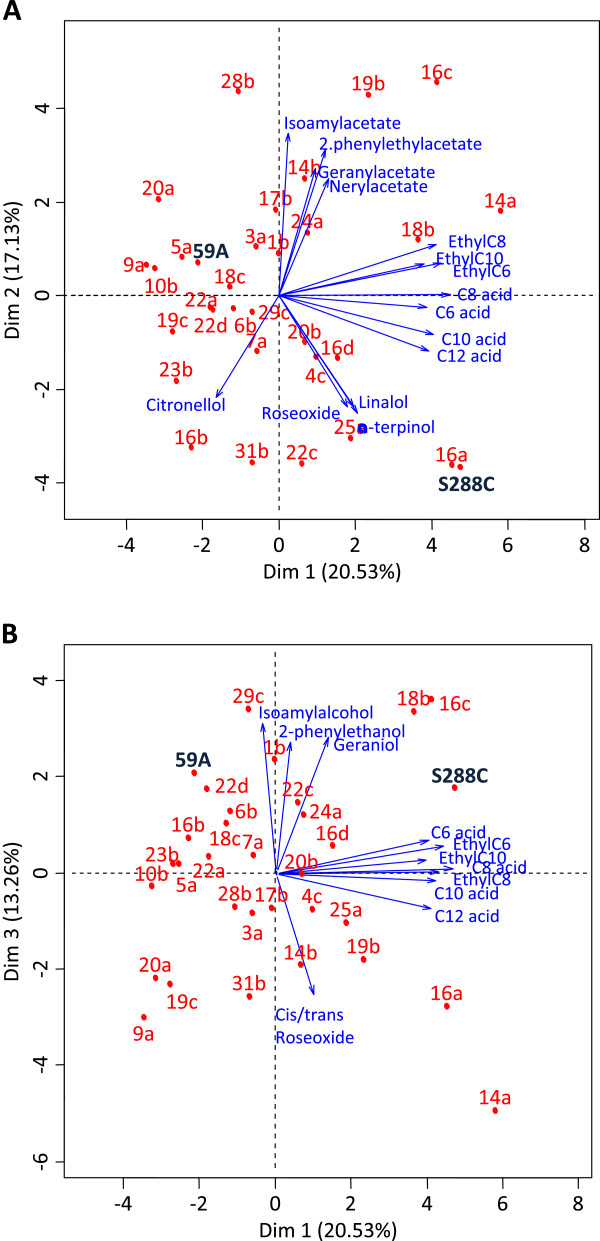
**Principal component analysis presenting the variability in the concentration of volatile compounds produced by the various segregants and parent strains (experiment A). A:** Components 1 and 2 representing 37.7% of the global variation; **B:** Components 1 and 3 representing 33.8% of the global variation. Segregants are indicated in red, aroma compound vectors are given in blue.

The fermentation kinetics of the 30 segregants presented significant diversity, from typical wine fermentations to clearly sluggish as observed for S288C (which presented the longest fermentation). Unlike the parental strains, several segregants presented a clear ability to flocculate. This resulted in large and significant diversity in the concentrations of volatile compounds at the end of the alcoholic fermentations. We measured a set of 27 compounds in experiment A, and 33 compounds in experiment B. We performed a principal component analysis to reduce the multidimensional data set of experiment A into three more informative dimensions (Figure 1). The first three axes explained 51% of the global variance (37.6% for axes 1 and 2 in Figure 1A and 33.7% for axes 1 and 3 in Figure 1B) and in this analysis the various compounds are grouped according to chemical family. Ethyl esters and medium chain fatty acids were correlated to the first axis, the various acetates correlated together with the second axis and 2-phenyl ethanol and isoamylalcohol were correlated to axis three. The representation of individual progeny strains in the factorial plan indicated a substantial diversity in the ability to produce volatile compounds. This was especially clear for acetates, as many strains were able to produce more acetates in the media than either of the two parents (S288C and 59A). In addition, some strains more than others metabolized geraniol into citronellol or into the high olfactive impact compound cis-rose oxide. This indicates that the characteristics of the yeast strain have a significant and variable impact on the grape aroma fraction. A similar picture was obtained from the analysis of experiment B.

### Genetic analysis of the volatile compounds production

From experiment A, heritability was estimated to be greater than 70% for 21 of 27 compounds, which included the grape aroma compounds geraniol, linalool, citronellol and geraniol acetate.

To identify QTL for these technological features, we performed a linkage analysis with the previously reported genotypes for these progeny [11]. The concentrations of most compounds obtained for the population of segregants did not follow normal distributions (Additional file 1), so we performed linkage analysis with both parametric and non parametric models. We identified four and six regions involved in variations in the production of different compounds in experiments A and B, respectively (Tables 1 and 2, respectively). However, additional factors may contribute to wine aroma production: flocculation is one [26]; and the presence of the *ABZ1*-S288C allele, which is responsible for large variations in fermentation kinetics [11], may also have an indirect effect. To overcome the potential effects of these factors, we performed a second linkage analysis taking these two factors into account as covariables in the model. This enabled us (i) to improve the significance for some QTL detected after a simple scan (such as for ethyl octanoate), (ii) to detect a genetic effect for additional compounds (ethyl hexanoate) of one region already found and (iii) to detect three and one additional genomic regions in experiments A and B, respectively, for other compounds. The effects of flocculation and of *ABZ1* allele on aroma production for each QTL are given as Additional file 2.

**Table 1:** **QTL analysis of volatile compounds produced during alcoholic fermentation** (**experiment A**, **geraniol 5mg**/**L**)

**Compounds**	**Localization**	**Single QTL scan**		**Flocculation as a covariable**		**Fraction of variation explained by the QTL**	**Heritability**
		**LOD**	**p**-**value**	**LOD**	**p**-**value**		
Isoamyl acetate							33
Isoamyl alcool							83
Ethyl hexanoate	Chr XIV 634-687			4.52	0.034	50.1	28
Ethyl octanoate							-
Ethyl decanoate							71
Ethyl myristate							70
2-phenylethyl acetate							80
2 phenyl ethanol	Chr VIII 422-469	3.23	0.04			39.1	99
Hexanoic acid							-
Octanoic acid							90
Decanoic acid							99
Myristic acid							97
Ethyl 9-decenoate							99
Nerolidol	Chr XII 675-704	8.28	<0.004			71.9	93
Farnesol							74
Ethyl 3-							
hydoxydecanoic acid							-
α-terpineol							22
Linalol							78
Citronellol	Chr XIII 290-342	3.69	0.033			43.3	78
Geraniol							98.
Nerol							-
Citronellyl acetate							79
Geranyl acetate							87
nerylacetate							63
Isobutanol							-
Cis-rose oxide	Chr I 21-55			4.59	0.02	51.1	62
Cis-rose oxide	Chr VII 47-85			4.27	0.04	48.6	
Trans-rose oxide							90
Cis/trans rose oxide ratio	Chr XIV 537-589	3.94	0.01			45.4	99

**Table 2:** **QTL analysis of volatile compounds produced during alcoholic fermentation** (**experiment B**, **Ambroset et al**. **2011**)

**Compound**	**Localization**	**Single QTL scan**		**Flocculation (1) or*ABZ1*(2) as a covariable**		**Fraction of variation explained by the QTL**
	(**coordinates in kb**)	**LOD**	**p**-**value**	**LOD**	**p**-**value**	
Ethyl octanoate	Chr XIII 255-305	3.80	0.044	4.86	0.007 (**2**)	46.4
Ethyl decanoate	Chr XIII 245-304			4.62	0.024 (**2**)	
Ethyl myristate	Chr XIII 230-290	3.92	0.010			47.6
2-phenyl ethanol	Chr XIV 657-702	4.01	0.022			48.3
Dodecanoic acid	Chr VII 332-370			5.45	0.039 (**1**)	54.2
Nerolidol	Chr XII 674-705	3.94	0.004			46.0
Isoamyl octanoate	Chr VIII 423-481	3.46	0.033			43.4
Methyl oleate	Chr XIII 234-285	3.79	0.029			46.4
Farnesol						
(E,E)-Farnesol						
(E,Z)- or (Z,E)-Farnesol	Chr II 593-646	4.23	0.018	4.7	0.022 (**2**)	50.2
trans-β-farnesene	Chr XII 711-750	3.45	0.050			
(Z,E)-α-farnesene	Chr XII 693	3.31	0.050			
*α-bisabolene*	*Chr XII 735*	*3*.*46*	*0*.*059*			
β-bisabolene	Chr XII 706-757	3.71	0.011	3.89		45.6
(E,E)-α-farnesene	Chr XII 675-704	3.47	0.044	3.62		43.5

Regions above the 0.05 threshold are indicated in italics. Only compounds with differences that are significant or close to the significance threshold are given.

For several compounds we were unable to identify any QTL despite a high heritability. This were the case for instance for isoamyl-alcohol and its acetate ester. In the case of isoamyl-alcohol, this might be due to two isomeric compounds (3-methyl-1-butanol and 2-methyl-1-butanol) both being involved. Nevertheless, we detected several regions involved in the diversity of the production of various compounds in the acid, alcohols ethyl ester and isoprenoid chemical families. As a whole, these metabolic QTL (mQTL) explained between 43 and 73% of the metabolite variation.

**Figure 2:**
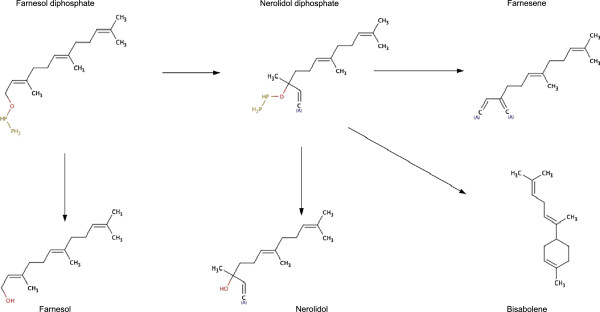
Chemical structure and relationship between farnesylpyrophosphate and derived compounds.

**Figure 3:**
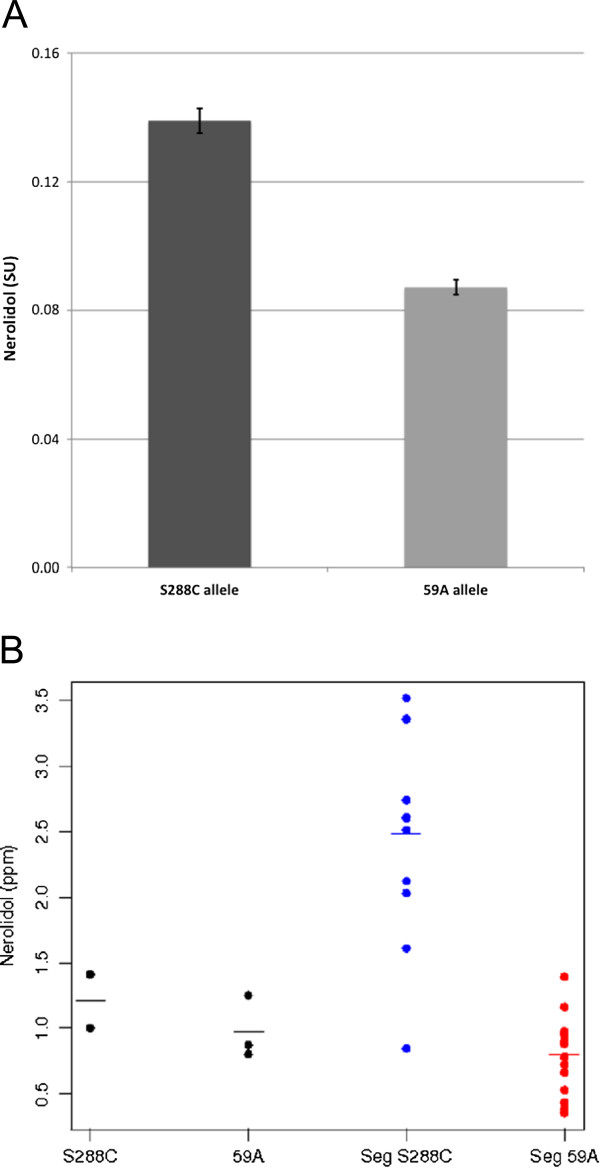
**A: Production of nerolidol by two hemizygote strains carrying only the 59A or the S288C** - ***PDR8 *allele.** Dark gray: S288C, Light Gray: 59A. Differences are highly significant (p value<0.001). For each experiment, three fermentations were performed as described for experiment B. **B**: Production of nerolidol by the different segregants, relative to that measured for the parental strains indicated as 59A and S288C. (Data from experiment A). Segregants carrying 59A allele of *PDR8* are indicated as Seg 59A, and segregants carrying S288C allele of *PDR8* are indicated as Seg S288C.

One region on chromosome XII was identified in both experiments with high Lod score values and explained as much as 46 and 72% of the variations in the production of nerolidol. The same region was identified for other isoprenoids characterized only in design B. Another region, on chromosome XIV, was also detected in both experiments and was associated with various phenotypes: ethyl hexanoate in experiment A and several compounds (including 2-phenyl ethanol and ethyl octanoate) in experiment B. The *ABZ1* gene which maps in this region has been reported to be responsible for variations in the rate of fermentation [11]. When *ABZ1* polymorphism was used as a covariable, we detected other QTL for more compounds indicating that *ABZ1* allelic variations may affect the production of several metabolites. These mQTL are scattered through the genome of strain S288C such that we did not detect a major region associated with all the compounds analyzed (Figures 2 and 3).

The other regions detected for several compounds were each found in only one experiment. These regions also explained a smaller part of the variations in the production of the various volatile metabolites despite a high heritability. Possibly, the production of most of these compounds is under multigenic control and the corresponding regions cannot be detected with such a small population of segregants.

In addition to compounds produced by yeast during alcoholic fermentation, we also studied the fate of geraniol, which is present in grape must at up to 3 mg/L [27]. We did not detect any region explaining variations in the metabolism of geraniol. However, we detected one QTL explaining 43% of the variations in the concentration of citronellol a compound produced from geraniol during alcoholic fermentation. The synthetic pathway for citronellol has not been clearly described and our results may indicate new targets to investigate. Two other QTL explained variations in the content of cis-rose oxide and in the ratio between cis and trans isomers of rose oxide. These QTLs may be technologically interesting as the two isomers of rose oxide present different olfactive thresholds.

#### Evaluation of the role of various candidate genes in the QTL

##### *PDR8* is responsible for variations in nerolidol production during alcoholic fermentation

The major QTL responsible for variations in the concentration of nerolidol in experiment A and of nerolidol, farnesene and bisabolene in experiment B maps to a short region of 20 kb containing 26 ORFs. Nerolidol, farnesene and bisabolene are all derived from farnesyl diphosphate, an intermediate in isoprenoid and ergosterol biosynthesis (Figure 2): at acidic pH, the instability of the diphosphate group leads to the release of farnesol and its isomer nerolidol.

It seemed likely that the gene involved in the modulation of nerolidol, farnesene and bisabolene production is involved in ergosterol biosynthesis or in farnesol/nerolidol transport because of the size of these molecules. One of the genes mapping in this region is *PDR8*, a transcription factor that modulates the expression of 16 genes [28] including transporters (*AZR1*, *PDR15*, *QDR2*, *YOR1*), a gene of the ergosterol biosynthesis pathway (*ERG8*), and enzymes involved in oxido-reduction processes (*CTT1*, *GTT2*, *YMR315w*). This transcription factor was clearly a good candidate. The nucleotide sequences of the *PDR8* genes in strains S288C and 59A show numerous single nucleotide differences. These SNPs generate five non-synonymous substitutions between the Pdr8p proteins in 59A and S288C.

To confirm the involvement of the *PDR8* gene in the observed phenotype, we compared two reciprocal hemizygotes between S288C and 59A containing only one of the parental alleles. These hemizygotes presented the different phenotypes observed in the population of segregants, with the enological *PDR8* allele of 59A leading to a lower production of nerolidol (Figure 3A). These results are in agreement with those obtained for the whole population (Figure 3B). However, the parental strain S288C produced less nerolidol than most of the segregants, indicating further interactions with the genetic background.

#### Characterization of PDR8 targets involved in the phenotype

**Figure 4:**
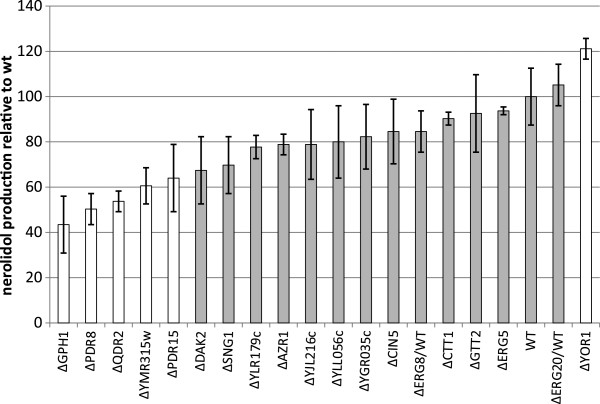
**Production of nerolidol by mutant strains deleted for *PDR8 *targets**, **reported relative to the production by the wild-type strain By4741.** The Dunnett test was used to compare the production by each deletant strain to that by the corresponding wild type strain (p-value <0.05). Each experiment was repeated at least twice. For By4742 ΔERG20/wt and BY4742 ΔERG8/wt the wild-type control strain is By4743. Bars in gray indicate that the production was not significantly different to that by the wt. White bars indicate that the production of nerolidol was significantly different from that by wt.

**Figure 5:**
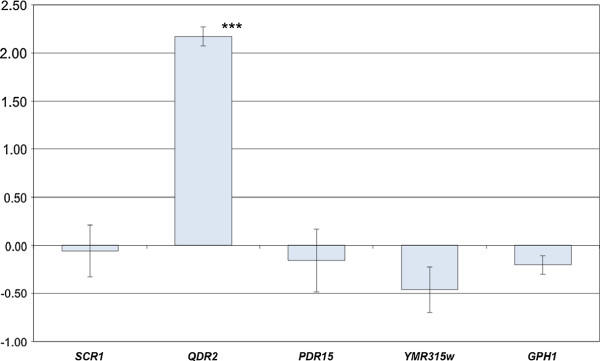
**Q-PCR analysis of the expression of *PDR8 *targets in 59A strains bearing 59A or S288C alleles during alcoholic fermentation. Differences in expression are given as fold ratio in comparison to 59A.** Only the expression of QDR2 was highly significantly different (pvalue <0.001) for both strains. Other differences were not significant.

To identify which of the targets of *PDR8* explain the observed variations in nerolidol production, we measured the production of nerolidol by the corresponding 16 deletant strains in the By4741 background (Figure 4). The deletion of *YOR1* led to an increase of nerolidol production whereas the deletion of four other *PDR8* target genes (*QDR2*, *PDR15*, *GPH1* and *YMR135W*) led to decreases of nerolidol production similar to that observed after the deletion of *PDR8*. The genes *QDR2* and *PDR15* encode transporters that may be involved in the export of nerolidol or derived compounds from the cell. The deletion of two other genes, *GPH1* and *YMR315W*, resulted in a similar reductions in nerolidol production indicating other possible mechanisms. *GPH1* is a glycogen phosphorylase required for the mobilization of glycogen, and *YMR315W* is an oxidoreductase enzyme thay may be involved in the reduction of farnesol (data not shown). *ERG8*, encoding mevalonate phosphate kinase, is an essential gene for isoprenoid and ergosterol biosynthesis, so it was not possible to conduct the appropriate tests with the deleted haploid strain. Deletion of only one copy of *ERG8*, in the diploid strain By4743, did not lead to any relevant change so we evaluated the effect of the overexpression of *ERG8*: no significant increase of the production of nerolidol was detected (data not shown). We did not detect any variation in the expression of *QDR2*, *PDR15*, *GPH1* and *YMR135W* associated with the *PDR8* allelic form reported in the experiment by Ambroset et al. [11], probably because of the high FDR rate. Therefore, we replaced the *PDR8* allele in strain 59A and we compared the expression of these four genes between the strains containing each of the two alleles of *PDR8*. Quantative PCR (Figure 5) indicated that only *QDR2* was more strongly expressed in the strain carrying the S288C-*PDR8* allele.

#### ABZ1 allelic variations affect production of 2-phenylethanol and ethyl esters by yeast during fermentation

**Figure 6:**
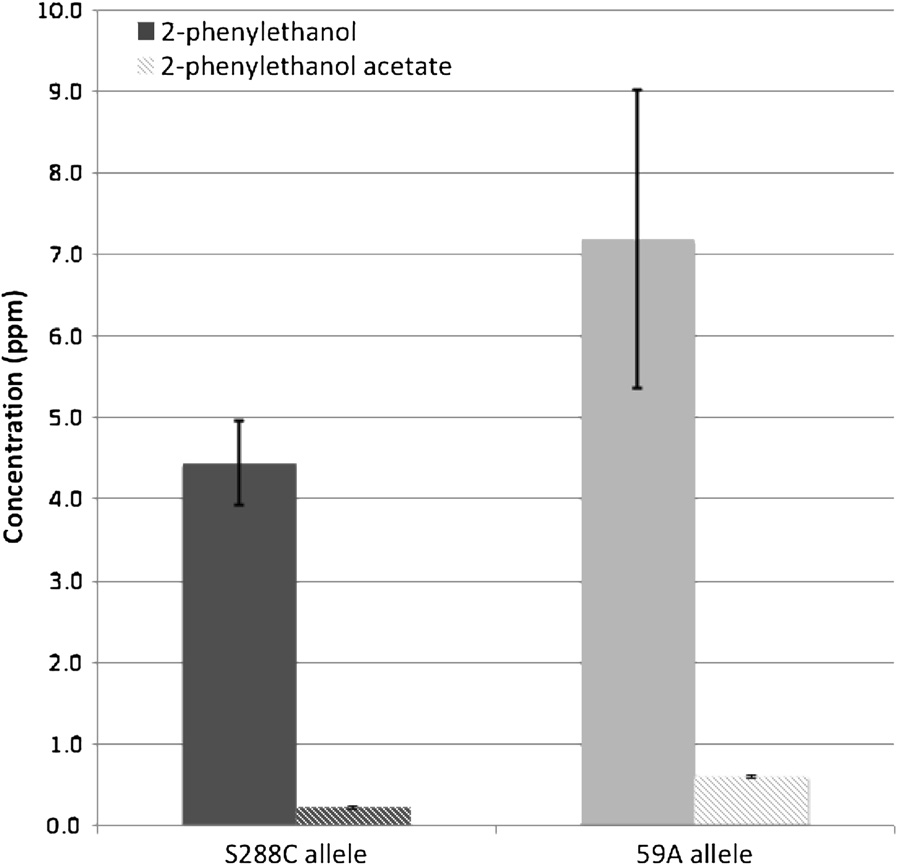
**Production of 2-phenylethanol and 2-phenyethylacetate by two hemizygote strains carrying only the 59A or the S288C – *ABZ1 *allele.** Fermentation was performed as described for experiment B. Dark gray: S288C. Light gray; 59A. Differences are highly significant (p value<0.001). Errors bars correspond to one SD.

The variations in the concentrations of 2-phenyl ethanol and of ethyl hexanoate esters were linked to another mQTL corresponding to a 33 kb region of chromosome XIV. This region overlaps a region involved in differences in fermentation kinetics due to allelic variations of the *ABZ1* gene [11]. There are five non synonymous mutations between the S288C and 59A alleles of *ABZ1*. We compared two reciprocal hemizygotes between strains S288C and 59A containing only one allele of each origin to confirm the role of this gene in 2-phenylethanol production. The hemizygote which carried the enological allele of 59A, produced more 2-phenylethanol than the hemizygote which carried the S288c allele (Figure 6) . The addition of 1 mg/L of p-aminobenzoic acid to the fermentation media suppressed the differences in the rates of fermentation of the two strains; it caused a reduction of only 15% of the difference in the production of 2-phenylethanol (Additional file 3: Table S3), but completely abolished the differences in 2-phenyl acetate production. Abz1p uses chorismate as a substrate, which is also one of the precursors of 2-phenylethanol synthesis. We tested for the effects of the two alleles on the concentration of the various compounds analyzed during mQTL analysis (Table 3). We observed significant effects on the concentrations of many volatile compounds, including ethyl esters, confirming the involvement of *ABZ1* in their variations. These results also validate the use of *ABZ1* as an additive covariable in the model used to search for mQTLs.

**Table 3:** **Effects of the different alleles of the ABZ1 gene on the concentrations of several fermentation compounds** (**relative units**)

**Compounds**	**S288C ABZ1**			**59A ABZ1**			**p**-**value**
**Isoamylacetate**	**0**.**521**	±	**0**.**015**	**1**.**011**	±	**0**.**045**	**0**.**006**
**farnesyl acetate**	**0**.**439**	±	**0**.**013**	**0**.**780**	±	**0**.**046**	**0**.**006**
Isoamyl octanoate	0.057	±	0.005	0.060	±	0.006	0.729
**Isoamyl decanoate**	**0**.**076**	±	**0**.**001**	**0**.**092**	±	**0**.**004**	**0**.**048**
Ethyl hexanoate	0.510	±	0.005	0.459	±	0.011	0.060
**Ethyl octanoate**	**3**.**349**	±	**0**.**107**	**2**.**613**	±	**0**.**065**	**0**.**006**
**Ethyl decanoate**	**3**.**846**	±	**0**.**065**	**4**.**317**	±	**0**.**055**	**0**.**028**
**Ethyl dodecanoate**	**0**.**709**	±	**0**.**005**	**1**.**334**	±	**0**.**057**	**0**.**006**
**Ethyl myristate**	**0**.**035**	±	**0**.**001**	**0**.**060**	±	**0**.**002**	**0**.**002**
**Ethyl hexadecanoate**	**0**.**205**	±	**0**.**020**	**0**.**328**	±	**0**.**015**	**0**.**040**
Ethyl octadecanoate	0.068	±	0.013	0.085	±	0.012	0.399
Ethyl 9-decenoate	0.011	±	0.000	0.010	±	0.001	0.738
Ethyl 4-hydroxybutanoate	0.017	±	0.001	0.038	±	0.007	0.090
Ethyl 3-hydroxyoctanoate	0.052	±	0.004	0.039	±	0.002	0.086
**Ethyl 3-hydroxydecanoate**	**0**.**133**	±	**0**.**010**	**0**.**086**	±	**0**.**011**	**0**.**008**
**Ethyl 9**-**hexadecenoate**	**0**.**142**	±	**0**.**015**	**0**.**456**	±	**0**.**010**	**0**.**002**
**2-phenylethyl acetate**	**0**.**221**	±	**0**.**011**	**0**.**599**	±	**0**.**022**	**0**.**015**
**2-phenylethyl hexanoate**	**0**.**035**	±	**0**.**002**	**0**.**031**	±	**0**.**002**	**0**.**001**
**2-phenylethyl octanoate**	**0**.**011**	±	**0**.**001**	**0**.**022**	±	**0**.**005**	**0**.**004**
**Acetic acid**	**0**.**069**	±	**0**.**004**	**0**.**059**	±	**0**.**023**	**0**.**016**
**Octanoic acid**	**0**.**450**	±	**0**.**018**	**0**.**558**	±	**0**.**049**	**0**.**034**
**Decanoic acid**	**1**.**671**	±	**0**.**068**	**2**.**092**	±	**0**.**095**	**0**.**067**
**Dodecanoic acid**	**0**.**474**	±	**0**.**030**	**0**.**823**	±	**0**.**018**	**0**.**013**
**methyl Oleate**	**0**.**172**	±	**0**.**012**	**0**.**445**	±	**0**.**020**	**0**.**014**
isobutanol	1.508	±	0.327	1.230	±	0.067	0.408
isoamyl alcohol	17.440	±	1.703	20.582	±	0.238	0.150
**1**-**octanol**	**0**.**031**	±	**0**.**001**	**0**.**042**	±	**0**.**003**	**0**.**036**
**2-phenyl ethanol**	**4**.**435**	±	**0**.**369**	**7**.**201**	±	**0**.**238**	**0**.**003**
Nerolidol	1.318	±	0.105	1.428	±	0.053	0.334
2,3-dihydrofarnesol	3.245	±	0.396	2.895	±	0.220	0.411
**farnesol**	**3**.**948**		**0**.**220**	**2**.**361**	±	**0**.**087**	**0**.**006**
Trans-β-farnesene	0.057	±	0.005	0.060	±	0.006	0.623
Trans-α-farnesene	0.034	±	0.003	0.037	±	0.006	0.609
Cis-β-farnesene	0.036	±	0.005	0.043	±	0.007	0.387
Cis-bisabolene	0.006	±	0.001	0.007	±	0.001	0.372

Compounds whose concentration varies significantly are given in bold. Mean of 3 triplicates +/- standard deviation.

#### PLB2 allelic variations may affect ethyl ester production

**Figure 7:**
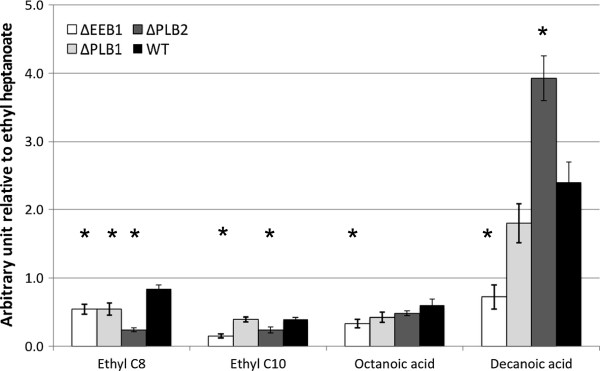
**Effects of the deletion of *PLB1***, ***PLB2***, **and *EEB1 *on the production of octanoic and decanoic acids and their corresponding ethyl esters**, **reported relative to the wildtype strain.** Fermentations were as for experiment B. White bars: ByΔ*EEB1*, Light gray bars: ByΔ*PLB1*, Dark gray bars: ByΔ*PLB2*, Black bars: By4741wild-type strain. The Dunnett test was used to compare the production by each deletant strain to that by the corresponding wild-type strain (p-value<0.05). *= results significantly different to wt [30-33] .

A 60kb region of chromosome XIII was linked with variations in the production of ethyl esters, and we identified two candidate genes with two allelic forms in this region: *PLB1* and *PLB2*. These genes code for phospholipase B which displays transacylase activity *in vitro*[29]. Plb1p in 59A presents some minor differences to that in S288C, whereas Plb2p of S288C carried a P378A substitution with respect to that in 59A. This proline residue is conserved in other *Saccharomyces* species and the mutation was not found in other available *S*. *cerevisiae* genome sequences. The Δ*PLB2*-By4741 strain produced much less octanoic ethyl ester than the control (Figure 7), and the difference was greater than that associated with deletion of *EEB1*, one of the key genes involved in the synthesis of decanoic ethyl ester [20]. These findings are consistent with the involvement of *PLB2* in this phenotype. Deletion of *PLB2* also led to a decrease in decanoic ethyl ester production and an increase of decanoic acid production, which was not observed after the deletion of *EEB1*[20].

#### Polymorphism of the various genes and adaptation

**Figure 8:**
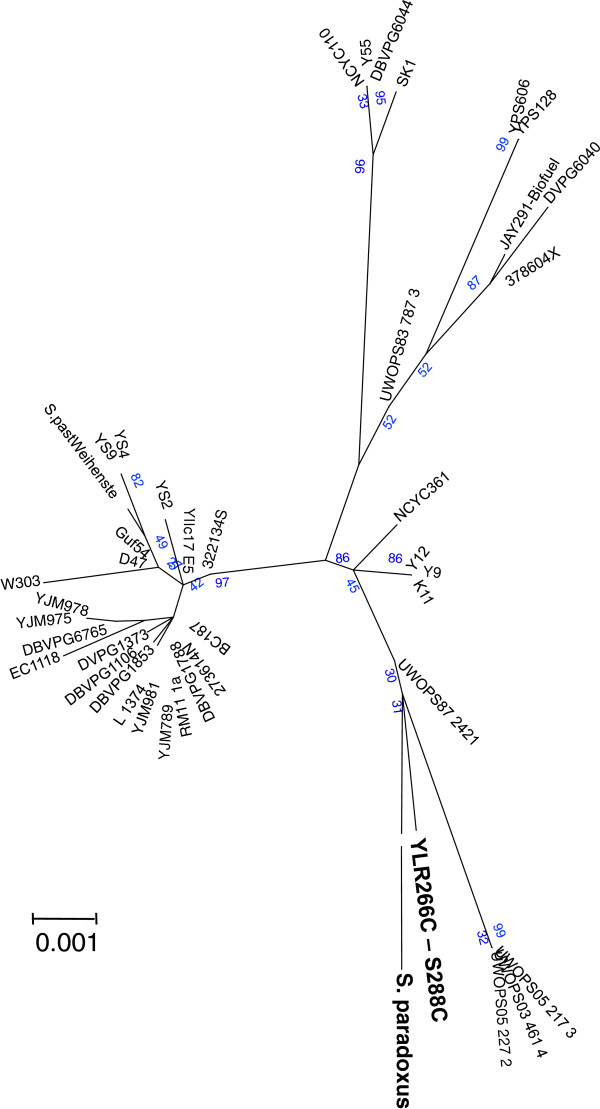
***PDR8 *molecular phylogenetic tree.** The evolutionary history was inferred by the maximum likelihood method based on the Kimura 2-parameter model and using 43 nucleotide sequences from the genome sequences available [30-33].

The two major QTL detected in this study, *PDR8* and *ABZ1*, show substantial polymorphism with numerous differences between the allelic forms in S288C and 59A. We investigated whether the differences between the alleles originated from the introgression from a specific lineage by comparing the corresponding alleles from other yeast genome sequences. The phylogeny (Figure 8) reveals that the *PDR8* allele of S288C is related to Malaysian or Asian alleles, and apparently one of the closest to its *S*. *paradoxus* ortholog; by contrast, the allele in 59A is a typical wine allele. As polymorphism may also result from specific adaptation, we performed a McDonald Kreitman test [34]. This test compares the ratio of nonsynonymous to synonymous polymorphism (intra species) to the ratio of nonsynonymous to synonymous divergence with the nearest species. This ratio is called the neutrality index (NI). An NI lower than one reflects a paucity of nonsynonymous polymorphism relative to nonsynonymous divergence, and is indicative of positive selection; an NI greater than one indicates negative selection of deletorious alleles driving divergence between species or balancing selection. This test was applied to a set of 15 *PDR8* alleles from strains isolated from various substrates and NI was 2.30, indicating a significant excess of non-neutral mutations (p value=0.009). This suggests that *PDR8* is subject to the accumulation of slightly deleterious mutations that are eliminated by negative selection during speciation, or alternatively that *PDR8* presents substantial diversity that might be associated with balanced selection resulting from specific adaptation to different niches.

**Figure 9:**
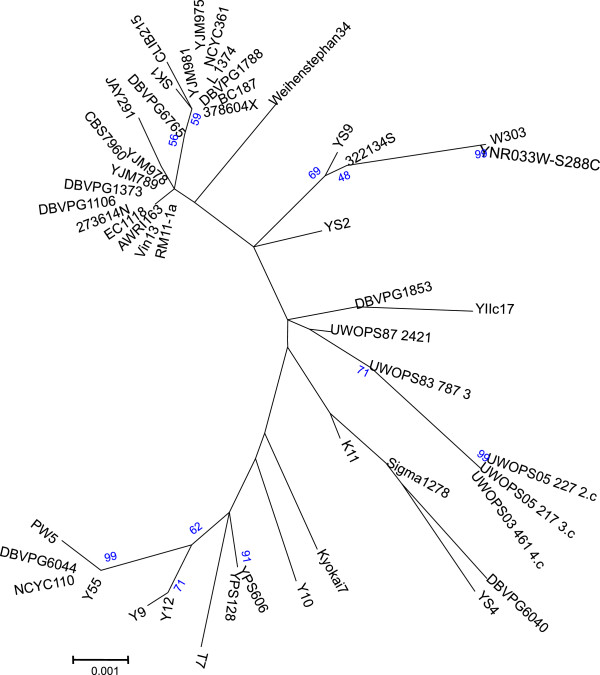
***ABZ1 *molecular phylogenetic tree.** The evolutionary history was inferred by the maximum likelihood method based on the Kimura 2-parameter model using 43 nucleotide sequences from the genome sequences available [30-33].

In contrast with *PDR8*, the overall phylogeny (Figure 9) revealed that the S288C *ABZ1* sequence is related to copies from clinical isolate 322134S and bread strains YS2 and YS4. However, the S288C allelic form of *ABZ1* is located at the end of a long branch such that it appears to be the result of the accumulation of numerous mutations. Similarly, the McDonald Kreitman test with a set of 15 *ABZ1* sequences from strains isolated from various substrates indicated an excess of non-neutral mutations (NI = 3.00, p value<10^-3^).

## Discussion

We report 13 regions linked to variations in the production of wine volatile compounds. This study is the first demonstration of the potential usefulness of QTL analysis for understanding the origin of the variations in the concentrations of wine aroma compounds and deciphering this “intricate lattice of chemical and biological interactions” [24]. It was not possible to detect QTL for all relevant compounds, despite high heritability. Presumably, the synthesis of many of these compounds is under multigenic control, such that the small size of our segregant population prevented exploration of their complexity. Until now, few key technological traits for alcoholic fermentation have been characterized [11,13,14,16].

Several of the QTL found here are related to terpenoids, which constitute a large family of compounds. They include monoterpenes, which with their corresponding alcohols present useful properties, such as fragrances (in essential oils) or variety aroma (in wines), and even antimicrobial and cancer chemopreventive properties [35]. In yeast, these compounds are synthesized through the mevalonic acid pathway from acetyl-coA, which is converted to isopentenylpyrophosphate (IPP) and its isomer dimethylallyl pyrophosphate (DMAPP), the building blocks of isoprenoids. The main product of this pathway is ergosterol, and geranyldiphosphate and farnesyl diphosphate are intermediate metabolites.

We did not detect any QTL explaining variations in residual geraniol. However, one QTL explained some of the variation in the concentrations of citronellol; this QTL maps to a region of chromosome XIII containing several candidate genes. We did not find a candidate explaining the variations in the concentration of cis-rose oxide in the media or in the ratio between the cis and trans isomers. This compound is significant to wine-making because of its high odor activity [36] and it has been shown recently that yeast can produce cis-rose oxide in wine [37].

We demonstrate that the alleles of *PDR8* found in S288C and 59A differently regulate the *QDR2* gene responsible for the release of nerolidol into the media. Farnesol and its isomer nerolidol arise from farnesyl diphosphate instability at low pH, like that in the yeast vacuole or in the exocellular medium [38]. Therefore, it is possible that the transporter Qdr2p is responsible of the export of either farnesyl diphosphate or of nerolidol.

Enological strains have a *PDR8* allele more divergent from *S*. *paradoxus* than that of S288C. The neutrality index [34] we calculated for this gene is incompatible with its neutral evolution: it presented a higher number of replacement polymorphisms than expected under neutral selection. This may be the result of adaptation to different niches or the results of isolation and multiple migrations as suggested by Aa et al. for *SSU1*[39]. The role of farnesol and nerolidol production by *S*. *cerevisiae* is not clear. Under the anaerobic conditions of wine fermentation ergosterol synthesis is blocked, however *ERG20* expression correlates with fermentation speed [11] even in a fermentation medium containing ergosterol (experiment B). The synthesis of farnesol diphosphate is essential for (i) the synthesis of other compounds including dolichol which is necessary for cell wall assembly [40,41], (ii) protein prenylation such as that of Skt5p [42] involved in chitin synthase activity, and (iii) ubiquinone synthesis which may be less important during fermentation. Furthermore, farnesol is a biologically active compound that at concentrations higher than 50 μM inhibits *S*. *cerevisiae* and *C*. *albicans* growth [43,44] and at lower concentrations is involved in quorum sensing by *C*. *albicans*[45,46].

In addition to the regions affecting terpenoid production, we also linked several other regions to variations in the concentrations of various volatiles. The *ABZ1* gene in one of these regions seems to have the widest effect as its allelic variations affected 2-phenylethanol and ethyl ester synthesis in experiment B, and explained as much as 50% of the variations in the concentration of ethylhexanoate in experiment A. The lower production of 2-phenylethanol was only partially restored by the addition of p-aminobenzoic acid to the fermentation media, and this suggests that this phenotype is not solely the consequence of the substantial effect of the *ABZ1*-S288C allele on fermentation speed via its impact on nitrogen metabolism [11]. The phylogeny of *ABZ1* was clearly different from that of *PDR8*: the *ABZ1*-S288C allele is located at the end of a long branch whose branch point is close to that of wine strains (relative to other origins). Again, the neutrality index [34] calculated for this gene was significantly higher than 1. Possibly, the allelic form of *ABZ1* has accumulated several deleterious mutations leading to a loss of activity. This feature may explain the very particular phenotypic behavior observed for S288C [47].

The two experimental sets we report generated complementary results. We observed effects of *PDR8* and *ABZ1* allelic variations in both experiments. Nevertheless, the impact of *ABZ1* polymorphism was much less pronounced in experiment A than B, as it explained only variation for ethylhexanoate production after correction for the flocculation effect. We also detected one additional region in experiment A only, and four in experiment B only; presumably the different sets of QTL identified in the two experiments reflect the effects of the different environmental conditions. By considering genes mapping in these regions, we identified *PLB2* as possibly involved in the variations of ethyl ester content in experiment B. This gene may have a role complementary to *EEB1* in the synthesis of ethyl esters during alcoholic fermentation.

## Conclusion

This study shows that linkage analysis can give valuable information about the metabolic pathways involved in production of volatile compounds in yeast, even with only a small population of segregants. We identified the involvement of the *PLB2* gene in the metabolism of ethyl esters, and evidenced the role of *PDR8* in the release of nerolidol into the media via the regulation of *QDR2* expression. We also showed that the weak activity of SC288C Abz1p allele leads to a lower production of many metabolites, including 2-phenyl ethanol, and that this effect was only partially relieved by supplementation with paminobenzoic acid. Other candidate genes are currently being evaluated (i.e. for citronellol synthesis). However, we could not find candidate genes in all regions detected, and despite a high heritability, we did not find any regions associated with the production diversity of many of the compounds considered. Possibly, a larger number of segregants is necessary for a more exhaustive analysis. Our results identify potential new targets for a marker-aided breeding strategy in yeast for the optimization of the production of volatile compounds during fermentation.

Interestingly, our genetic analysis revealed the particular evolution of the *PDR8* gene. This may reflect a specific adaptation to wine fermentation conditions, but raises questions about the role of farnesol and nerolidol for *S*. *cerevisiae* during fermentation.

## Methods

### Strains, growth conditions, and fermentation conditions

The two parental *Saccharomyces cerevisiae* strains studied were the standard strain S288c (MAT**a**; SUC2; gal2) and a haploid derivative of the industrial wine strain EC1118 (HO/ho), herein referred to as 59A (MATa; ho). This strain is prototrophic and has fermentation properties similar to the diploid strain EC1118. The population of 30 segregants obtained from these two parental strains used for QTL analysis have been genotyped after hybridization on high density olignonucleotide microarrays Affymetrix YGS98 oligoarrays.

The strains BY4742 (MAT**a**; his3Δ1; leu2Δ 0; lys2Δ 0; ura3Δ 0) and BY4742**Δ**ABZ1 (Mat**a**; his3Δ 1; leu2Δ 0; lys2Δ 0; ura3Δ 0; YNR033w::kanMX4), and BY4742 (MAT**a**; his3Δ 1; leu2Δ 0; lys2Δ 0; ura3Δ 0) and BY4742**Δ**PDR8 (Mat**a**; his3Δ 1; leu2Δ 0; lys2Δ 0; ura3Δ 0; YNR033w::kanMX4) were used for hemizygous constructions.

Allelic replacement at *PDR8* in 59A was obtained in three steps: 1) deletion of *PDR8* from 59A using the hphMX4 cassette for hygromycin resistance (pAG32). Primers for cassette amplification and verification were obtained form Euroscarf. 2) preparation of a replacement cassette containing *PDR8*-loxP-kanMX4-loxP by the insertion of loxP-KanMX4-loxP into the terminator of *PDR8* in strain S288c (primers are given in Additional file 4: Table S4). 3) replacement of the hphMX4 cassette from 59A *PDR8Δ*::*hph* with the *PDR8*-loxP-kanMX4-loxP replacement cassette from S288c and selection on YPD containing G418 (200 μg.l^-1^). The loss of hphMX4 cassette was verified by PCR and the absence of growth on hygromycin.

YPD medium was used for precultures at 28°C for 24h in 125 mL flasks with shaking.

Synthetic MS300 medium, which mimics a natural must [48] and [11], was used for fermentation experiments. The first experimental design mimicked white wine fermentation (20°C, low lipid content and containing sitosterol; experiment A). Geraniol, one of the key aroma compounds found in Gewürztraminer wine, was added to study its metabolism during alcoholic fermentation. We also analyzed the production of volatile compounds during fermentation as described in Ambroset et al 2011, which differed by the higher lipid content of the synthetic must and fermentation temperature 28°C (Table 4; experiment B). In some experiments, p-aminobenzoic acid was added to the fermentation media at 1 mg/L to study the effect of *ABZ1* alleles in the hemizygotes.

**Table 4:** **Differences in the two experimental designs used in this work** (**adapted from**[48])

**Experimental design**	**A**	**B **[11]
Temperature	20°C	28°C
Stirring	no	yes
Fermentation volume	150 mL	1 L
Anaerobic factors for 1L	Tween 5 μL	Tween 0.5 mL
	Oleic acid 0.05 μg	Oleic acid 5 μg
	Sistosterol: 15 μg/L	Ergosterol 1500 μg/L
Geraniol content (mg/L)	5	0
Number of fermentations	2	1

Fermentations were performed in 250 mL flasks equipped with airlocks to maintain anaerobiosis without stirring (design A) and in 1 L fermenters with constant stirring (design B). Small flask fermentations were weighed twice daily and stopped as soon as the daily loss was less than 1% of the expected total loss.

### Volatile compounds analysis

Wine aroma compounds were analyzed by the Stir Bar Sorptive Extraction method [49] adapted to our laboratory conditions, with a 1 μL injection volume. The analyses were performed with an Agilent 6890N gas chomatograph equipped with an Agilent 7683 automatic liquid sampler coupled to an Agilent 5975B inert MSD (Agilent Technologies). The gas chomatograph was fitted with a DB-Wax capillary column (60 m × 0.32 mm i.d. × 0.50 μm film thickness, J&W Scientific) and helium was used as carrier gas (1 mL min^-1^ constant flow). The GC oven temperature was programmed without initial hold time at a rate of 2.7°C min^-1^ from 70°C to 235°C (hold 10 min). The injector was set to 250°C and used in pulsed splitless mode (25 psi for 0.50 min). The temperatures of the interface, MS ion source and quadrupole were 270°C, 230°C and 150°C, respectively. The mass spectrometer was operated in electron impact ionization mode (EI, 70 eV) and the masses were scanned over a m/z range of 29 – 300 amu. Agilent MSD chemStation software (G1701DA, Rev D.03.00) was used for instrument control and data processing. The mass spectra were compared with the Wiley’s library reference spectral bank

The following compounds were analyzed: isoamyl alcohol, isoamyl acetate, isobutanol, 2-3 butanediol, 2-phenylethyl acetate, ethyl hexanoate, ethyl octanoate, ethyl decanoate, ethyl dodecanoate, ethyl myristate, ethyl palmytate, ethyl laurate, 2-phenyl ethanol, hexanoic acid, octanoic acid, decanoic acid, dodecanoic acid, ethyl 9-decenoate, isoamyl octanoate, 2-phenyl ethyl hexanoate, 2-phenylethyl octanoate, 2-phenylethyl decanoate, ethyl 4 hydroxy butanoate, ethyl 3-hydroxydecanoate, ethyl 3-hydroxyoctanoate, nerolidol, farnesol, 2-3 dihydro farnesol, (E, Z)- or (Z, E)- farnesol (A), (E, E)- farnesol (A), farnesyl acetate, isoamyl octanoate, isoamyl decanoate, isoamyl dodecanoate, methyl oleate, trans-β-farnesene, (Z, E)-α-farnesene, α-bisabolene, ß-bisabolene, (E, E)-α-farnesene, α-terpineol, linalol, citronellol, geraniol, nerol, citronellyl acetate, geranyl acetate, neryl acetate, cis-rose oxide, trans-rose oxide.

### Statistical and QTL analysis

Heritability was calculated according to the method of Brem et al. [50]. Statistical analyses were performed using R software version 2.13.1 [51]. QTL analysis was done for each phenotype of the two datasets (experiments A and B) using the genetic map of 1834 markers genotyped previously [11]. The distribution of each phenotype was verified using a Shapiro-Wilk normality test: the normality of the distribution was rejected for 17 of the 29 compounds analyzed in experiment A and 30 of 40 in experiment B, for a threshold of 0.05

As the distribution of most phenotypes was not normal and due to the small sample size, linkage analysis was performed using both parametric and non-parametric models to evaluate the robustness of the parametric model. The parametric model consists of a linkage analysis performed using a normal model with the Haley-Knott regression method implemented in the R/qtl package [52,53]. As the results of the two analyses were concordant, only the normal analysis is presented.

To overcome the potential effects of flocculation [26] and of the presence of the *ABZ1*-S288C allele which provokes large variations in fermentation kinetics [11], we performed a second linkage analysis using a normal model with the Haley-Knott regression method, first with flocculation as an interactive covariate, and then with the *ABZ1* marker (Chr 14, position 689.4 kb) as an additive covariate. For these regions, a significant effect was indeed observed for both flocculation and the *ABZ1*-specific markers.

For the three models and the two datasets analyzed, logarithm of odds (LOD) scores were computed for each marker every 2.5cM. An interval estimate of the location of each QTL was obtained as the 1-LOD support interval. The LOD significance threshold was estimated after permutation tests that were replicated 1000 times. The percentage of variance explained by each QTL was estimated from a drop-one-term analysis of results in the global model.

### Q-PCR analysis of the expression of PDR8 targets after allelic replacement

Fermentations (900 mL of MS300 medium) were performed in triplicate with strains 59A and S288C-*PDR8* 59A, and cells were sampled when 70% of the glucose had been fermented. RNA was extracted with trizol as described previously [54]. cDNA was produced by reverse transcription and a 1 in 25 dilution of the resulting cDNA was used for the realtime PCR assays with gene-specific primers and Strategene’s Brilliant II SYBR Green QPCR Master Mix (Santa Clara, CA) and an ABI7300 QPCR machine. Expression levels were measured relative to those of *UBC6* and *SCR1*, both giving similar results.

### Sequence analysis and phylogeny

The comparison of the sequences of the 59A and S288C genomes and the differences between them can be found at http://genome.jouy.inra.fr/genyeastrait/[11].

To infer the evolutionary history of *ABZ1* and *PDR8*, we collected their sequences from genomes available at SGD (http://www.yeastgenome.org/). All uncompleted or frameshift-containing sequences where discarded from this set. The phylogenies were inferred with MEGA [55] by the Maximum Likelihood method based on the Kimura 2-parameter model [56]. The trees with the highest log likelihood are shown. The trees are drawn to scale, with branch lengths proportional to the number of substitutions per site. The significance of the Neutrality Index [34] test was calculated using the http://bioinf3.uab.cat/mkt/MKT.asp website.

The list of the sequences used for the two analyses is given in supplementary data (Additional file 5).

## Competing interests

The authors declare that they have no competing interests.

## Author’s contribution

DS: performed fermentation (experiment A), aroma and QTL analysis , candidate gene search and validation (*PDR8*, *PLB2*, …). DS wrote a first draft of the manuscript. CA produced the strain set and performed fermentations (experiment B). CB performed allelic replacement for S288c- *PDR8* 59A strain fermentations and QPCR expression analysis. PC, analyzed aroma compounds. PD built *ABZ1* hemizygote strains and performed fermentations. IS, performed statistical analysis (QTL). JLL performed phylogenic analysis and tests. CE, BB, FK, JLL conceived the study ,designed and coordinated the research JLL wrote the manuscript. All authors analyzed the data. All authors read and approuved the final manuscript.

## Supplementary Material

Additional file 1 Distribution of the different phenotypes for the population of segregants and parental strains. Click here for file

Additional file 2 Impact of flocculation and ABZ1 on aroma production at the different QTL and variablity of fermentation length. Click here for file

Additional file 3: Table S3 Effect of the addition of p-aminobenzoic acid on the production of 2phenylethanol and 2-phenylethanolacetate. Click here for file

Additional file 4: Table S4 Primers used in this study. Click here for file

Additional file 5 Sequenced used for McDonald Kreitman test. Click here for file
