# Supplementary material for: QTL mapping of the production of wine aroma compounds by yeast
Source: BMC Genomics. 2012 Oct 30;13:573. doi: 10.1186/1471-2164-13-573 (PMC3575298; doi:10.1186/1471-2164-13-573)
Supplement: Additional file 1 — Distribution of the different phenotypes for the population of segregants and parental strains. [file 1471-2164-13-573-S1.pdf]

Figure S1: Distribution of the different phenotypes for the population of segregants and parental strains

April 30, 2012

- parent S288C: black circle
- parent 59A: white circle

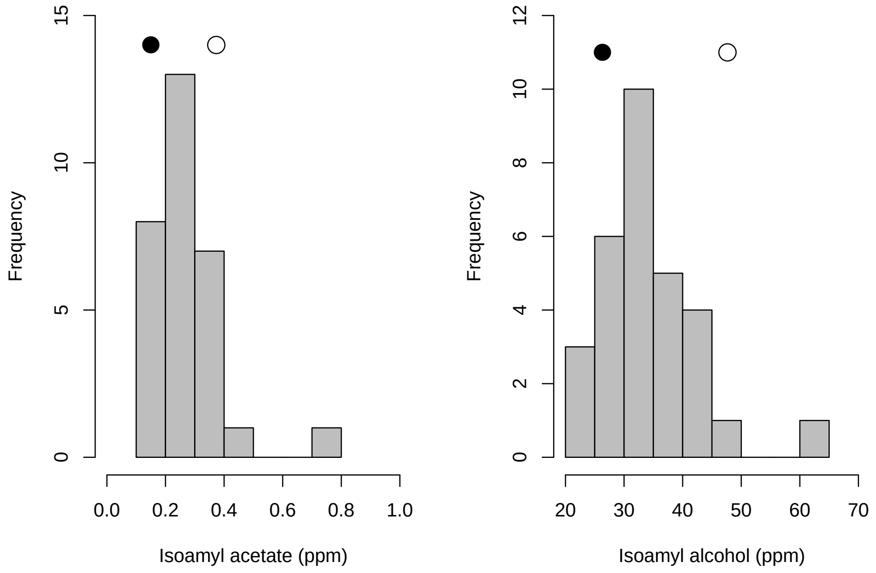

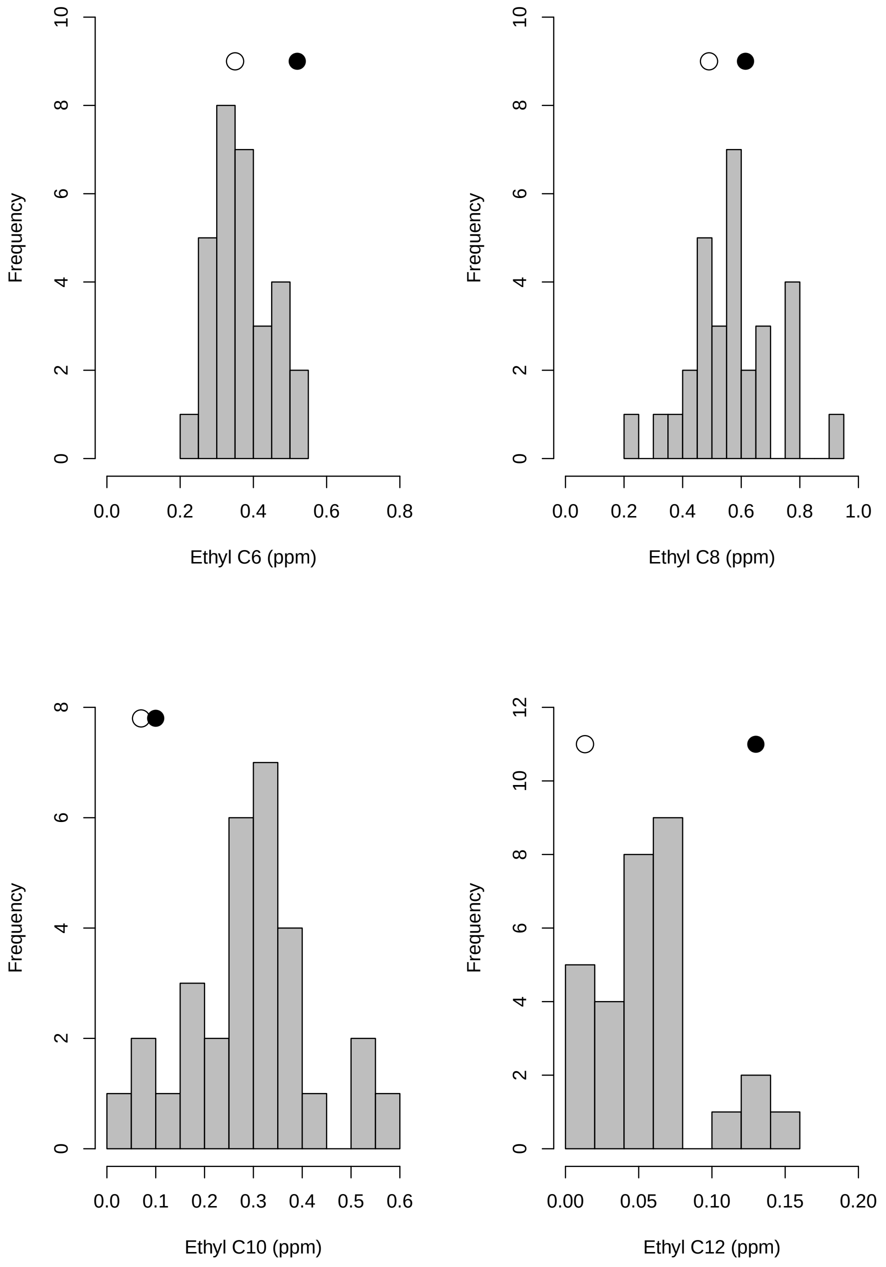

Frequency
Ethyl C6 (ppm)
Ethyl C8 (ppm)
Ethyl C10 (ppm)
Ethyl C12 (ppm)

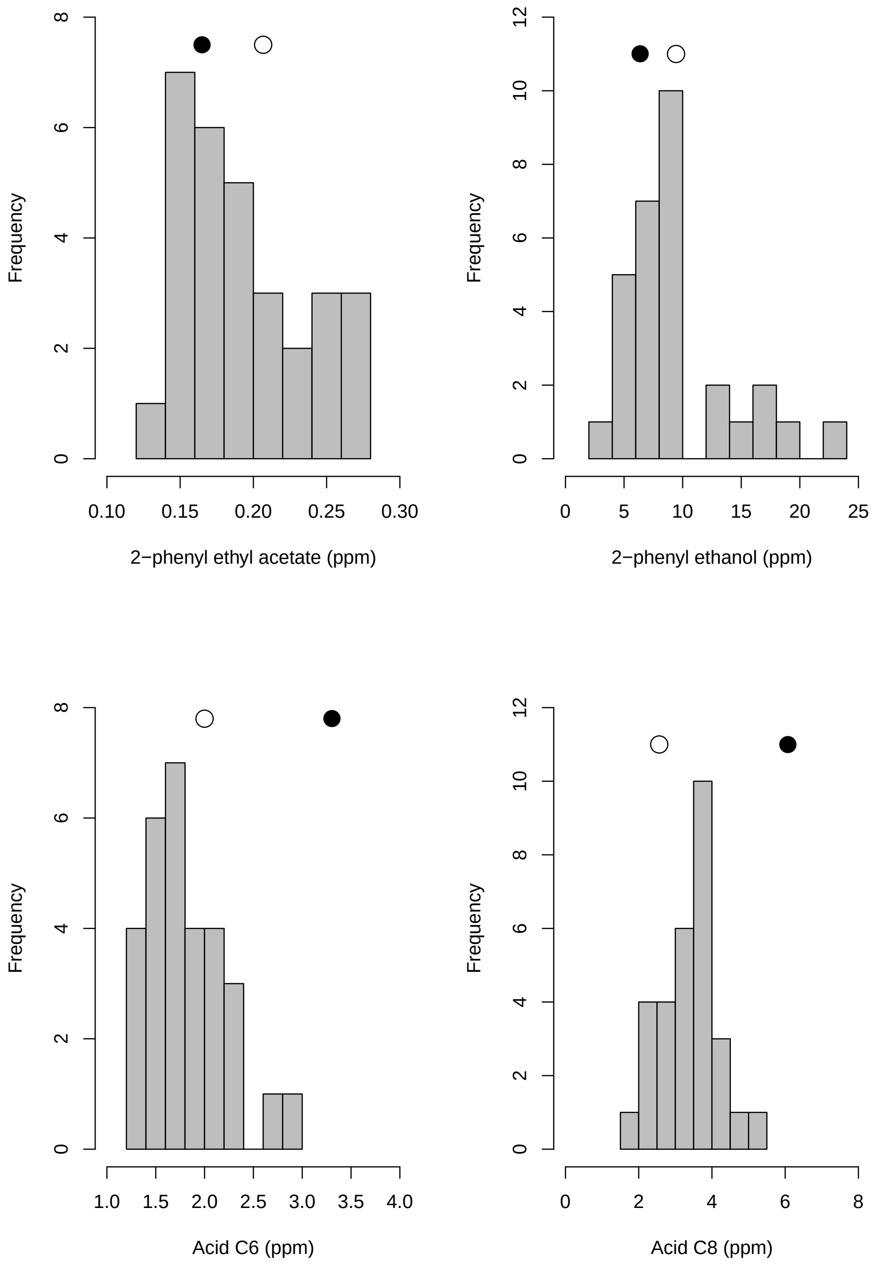
Frequency
2−phenyl ethyl acetate (ppm)
2−phenyl ethanol (ppm)
Acid C6 (ppm)
Acid C8 (ppm)

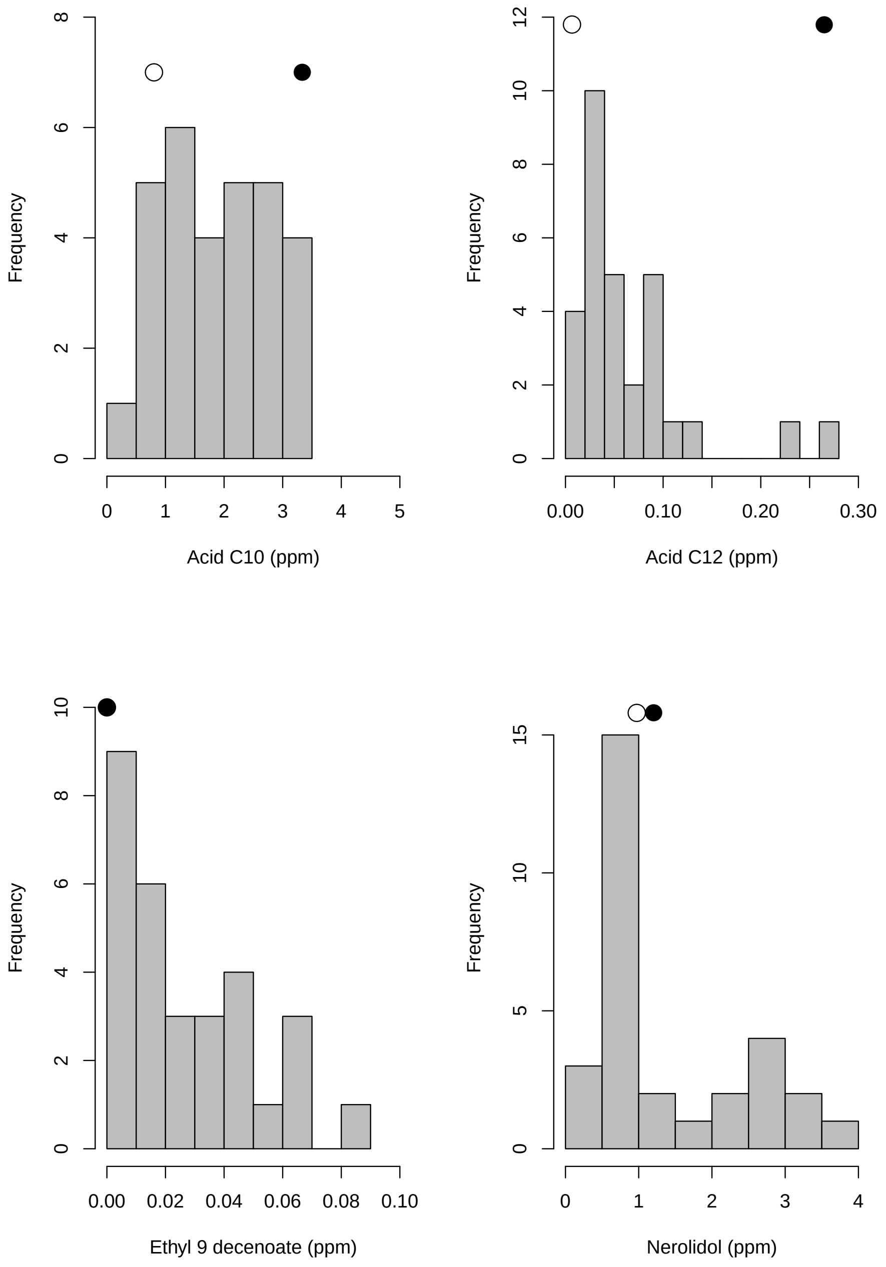
Frequency
Acid C10 (ppm)
Frequency
Acid C12 (ppm)
Frequency
Ethyl 9 decenoate (ppm)
Frequency
Nerolidol (ppm)

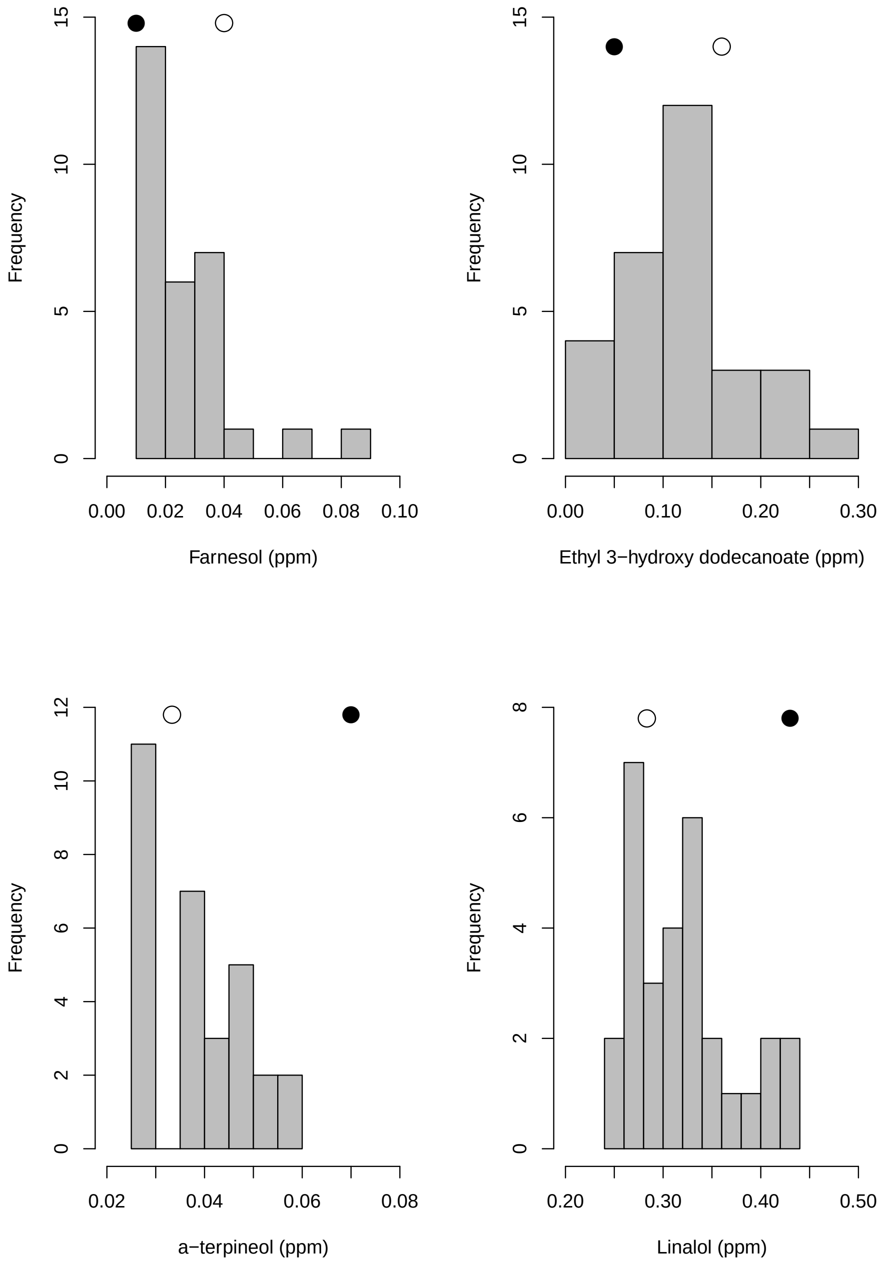
Frequency
Farnesol (ppm)
Ethyl 3−hydroxy dodecanoate (ppm)
a−terpineol (ppm)
Linalol (ppm)

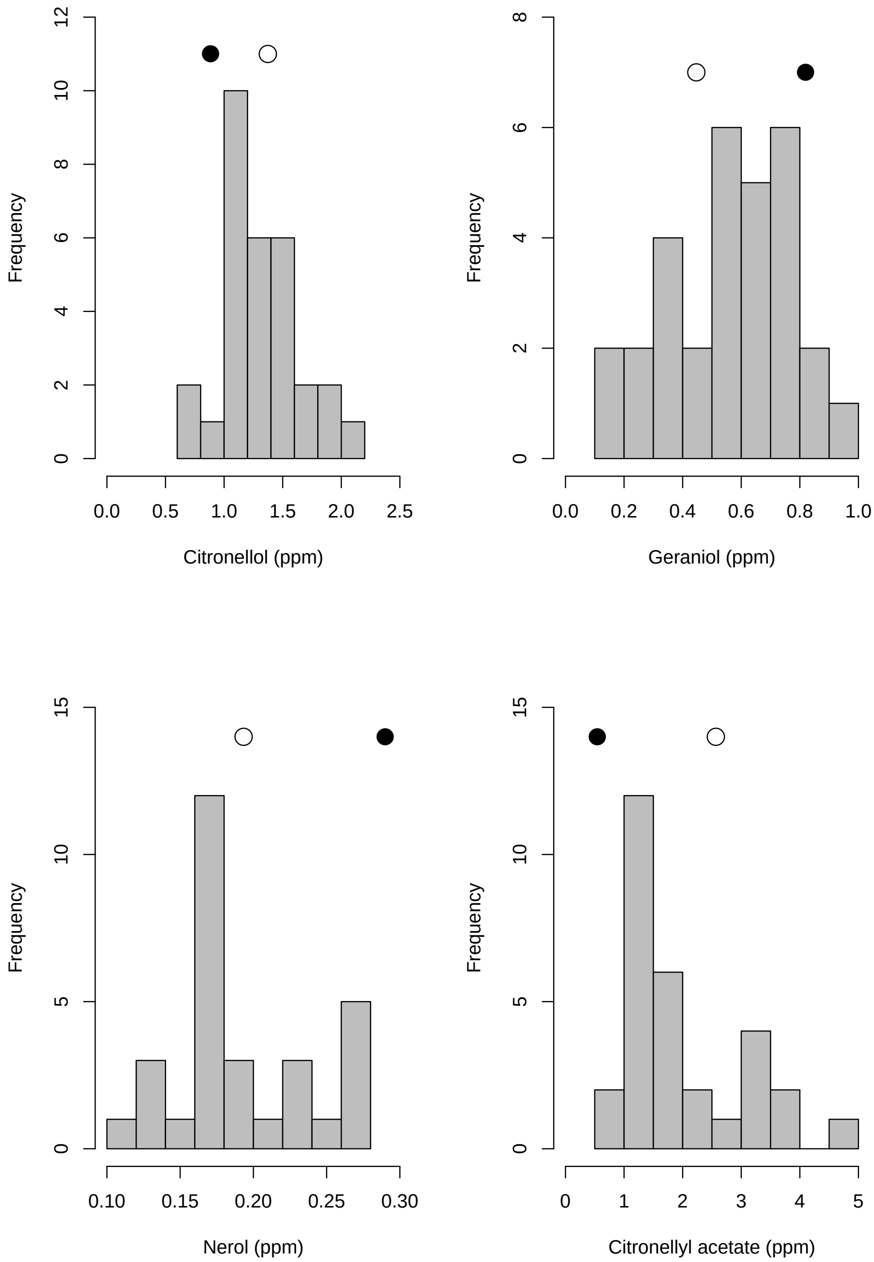

Frequency
Citronellol (ppm)
Geraniol (ppm)
Nerol (ppm)
Citronellyl acetate (ppm)

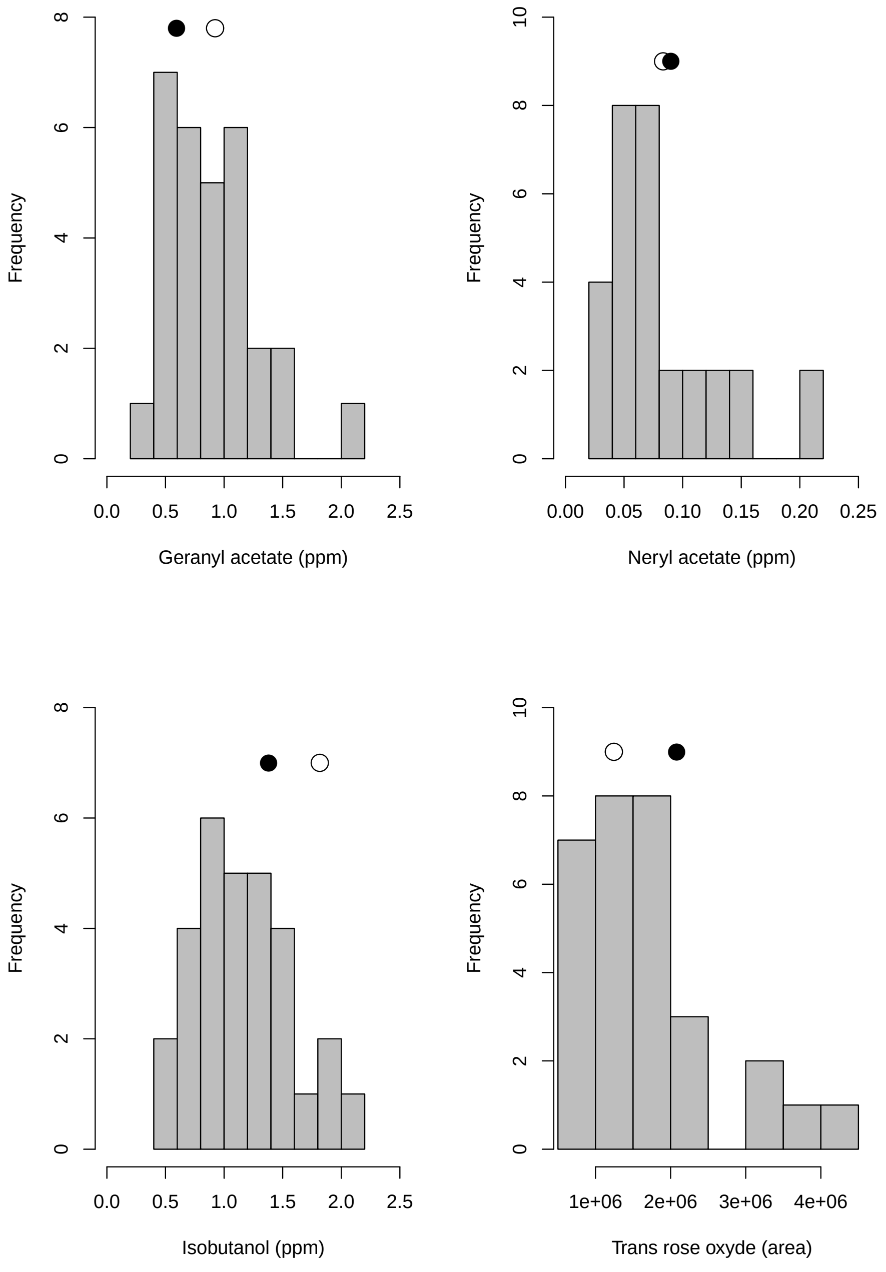
Frequency
Geranyl acetate (ppm)
Frequency
Neryl acetate (ppm)
Frequency
Isobutanol (ppm)
Frequency
Trans rose oxyde (area)

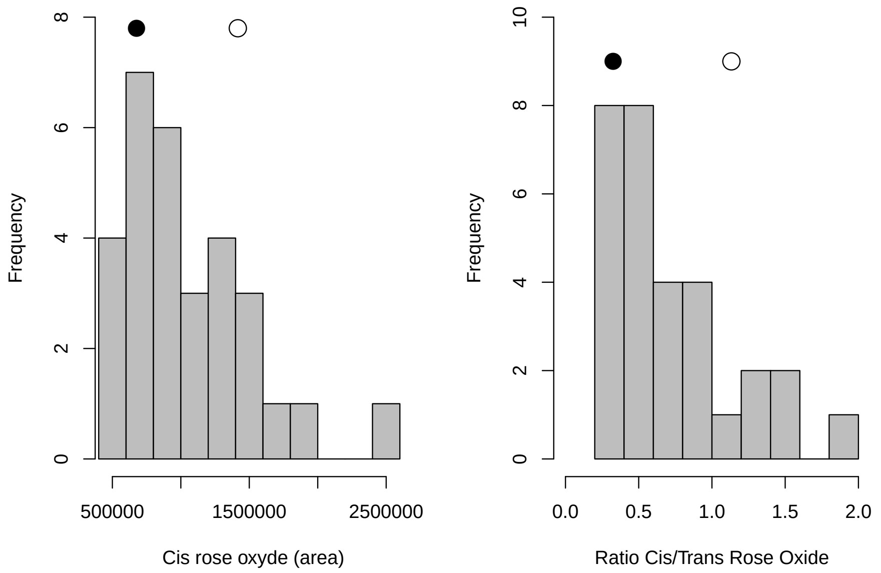
Frequency
Cis rose oxyde (area)
500000
1500000
2500000
Ratio Cis/Trans Rose Oxide
0.0
0.5
1.0
1.5
2.0
