# Supplementary material for: QTL mapping of the production of wine aroma compounds by yeast
Source: BMC Genomics. 2012 Oct 30;13:573. doi: 10.1186/1471-2164-13-573 (PMC3575298; doi:10.1186/1471-2164-13-573)
Supplement: Additional file 2 — Impact of flocculation and ABZ1 on aroma production at the different QTL and variablity of fermentation length. [file 1471-2164-13-573-S2.pdf]

Experiment A : Impact of flocculation on aroma production (additive model with interaction)

1- Effect of flocculation and locus Chr XIV :660000 on ethyl hexanoate production by segregants containing S288C or 59A allele (non flocculant strains: 0 in red, flocculant strains : 1, in blue). Error bars correspond to one SD.

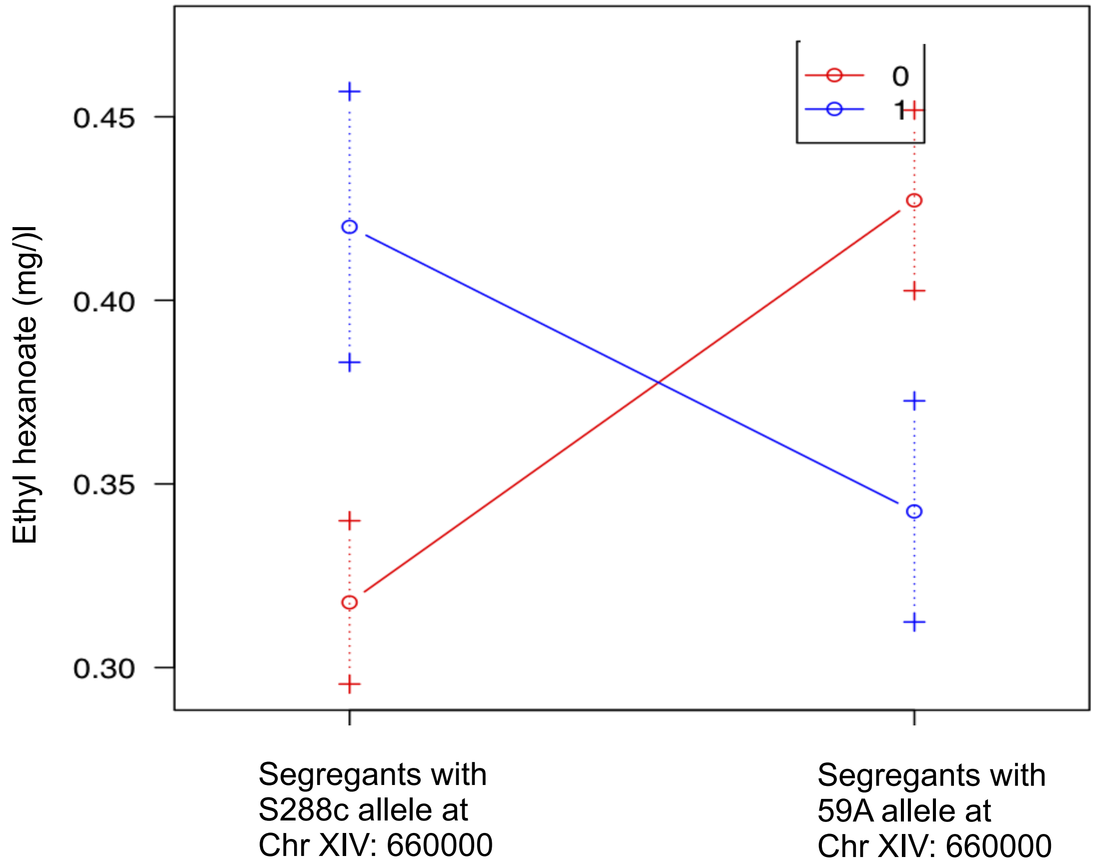

Results of QTL analysis with Haley-Knott regression
(Normal model, 30 segregants flocculation)

		Mean	SE	P value
Flocculation		0.01673	0.02360	0.0005
Marker at position Chr XIV 660000		0.13698	0.03246	0.0001
interaction		-0.31184	0.06908	0.0001

2- Effect of flocculation and locus chrI:38000 on the concentration of cis-roseoxide measured at the end of fermentation. Results are given for segregants containing S288C or 59A allele (non flocculant strains: 0 in red, flocculant strains : 1, in blue) in arbitrary units (au). Error bars correspond to one SD.

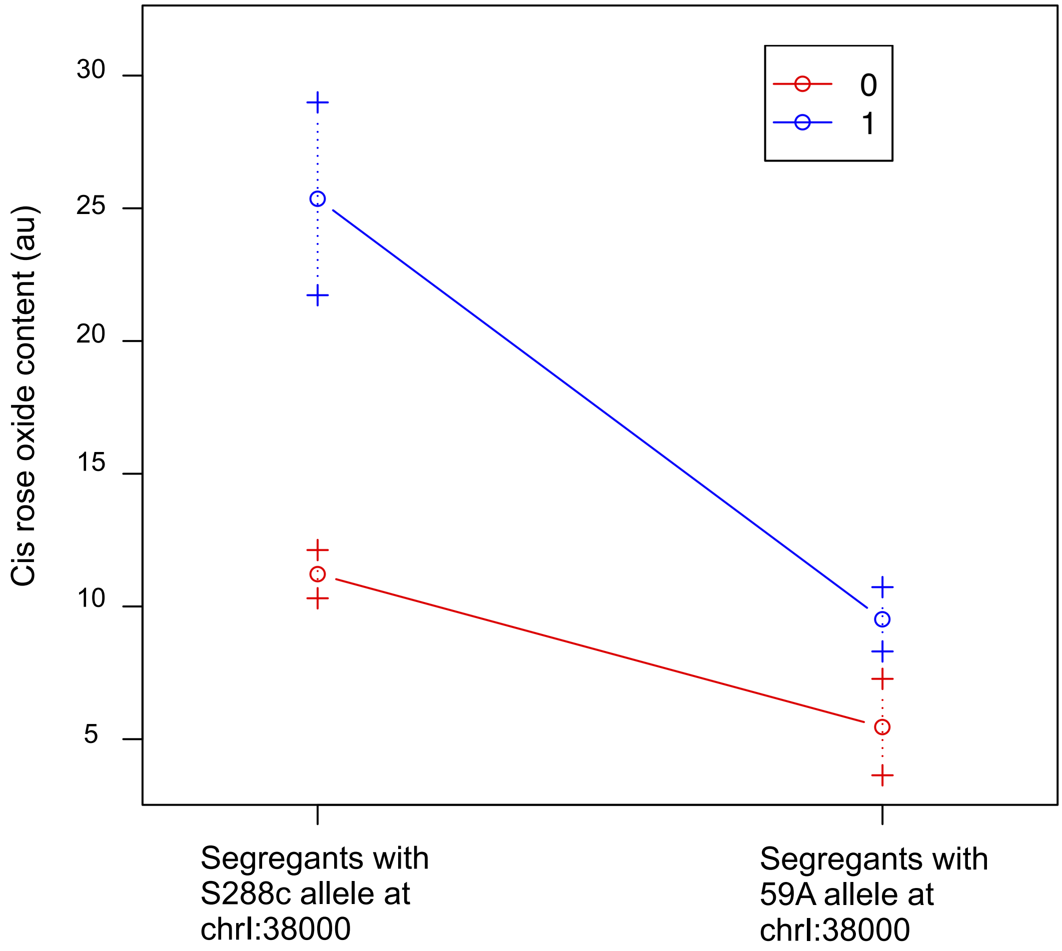

Results of QTL analysis with Haley-Knott regression for QTL (Normal model, 30 segregants)

	Mean (au)	SE	P value
Flocculation	9.10	9.9	0.0009
marker at chr I : 38000	-5.76	2.11	0.0001
interraction	-10.09	4.21	0.024

3- Effect of flocculation and locus Chr VII:66000 on the concentration of cis-roseoxide measured at the end of fermentation. Results are given for segregants containing S288C or 59A allele (non flocculant strains: 0 in red, flocculant strains : 1, in blue) in arbitrary units (au). Error bars correspond to one SD.

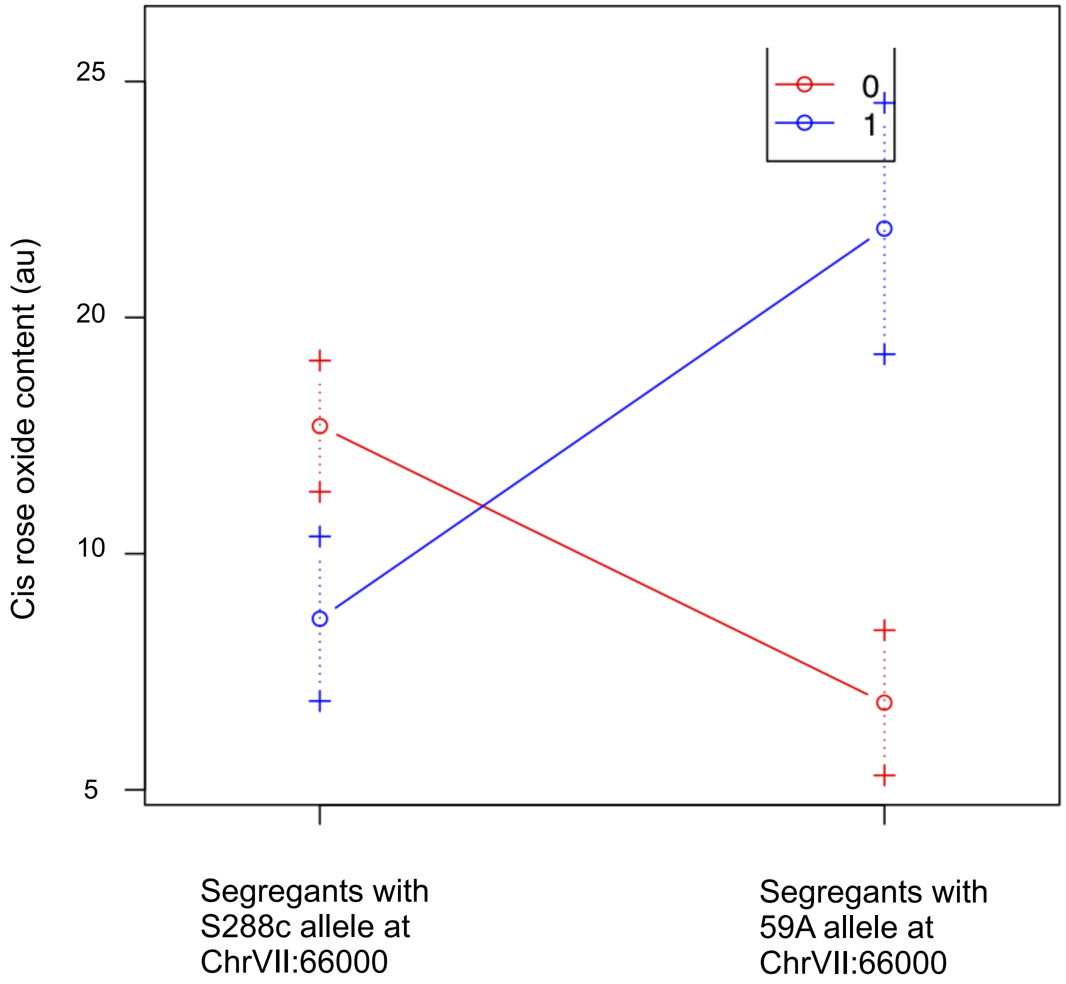

Results of QTL analysis with Haley-Knott regression for QTL (Normal model, 30 segregants)

		Mean (au)	SE	P value
Flocculation		9.10	9.9	0.0009
marker at Chr VII 66000		-5.76	2.11	0.0001
interraction		-10.09	4.21	0.024

2- Effect of *ABZ1* locus and locus Chr XIII:280000 on ethyl-decanoate production by segregants containing S288C or 59A allele (S288C-ABZ1allele : AA in red, 59A-ABZ1allele: AB in blue)

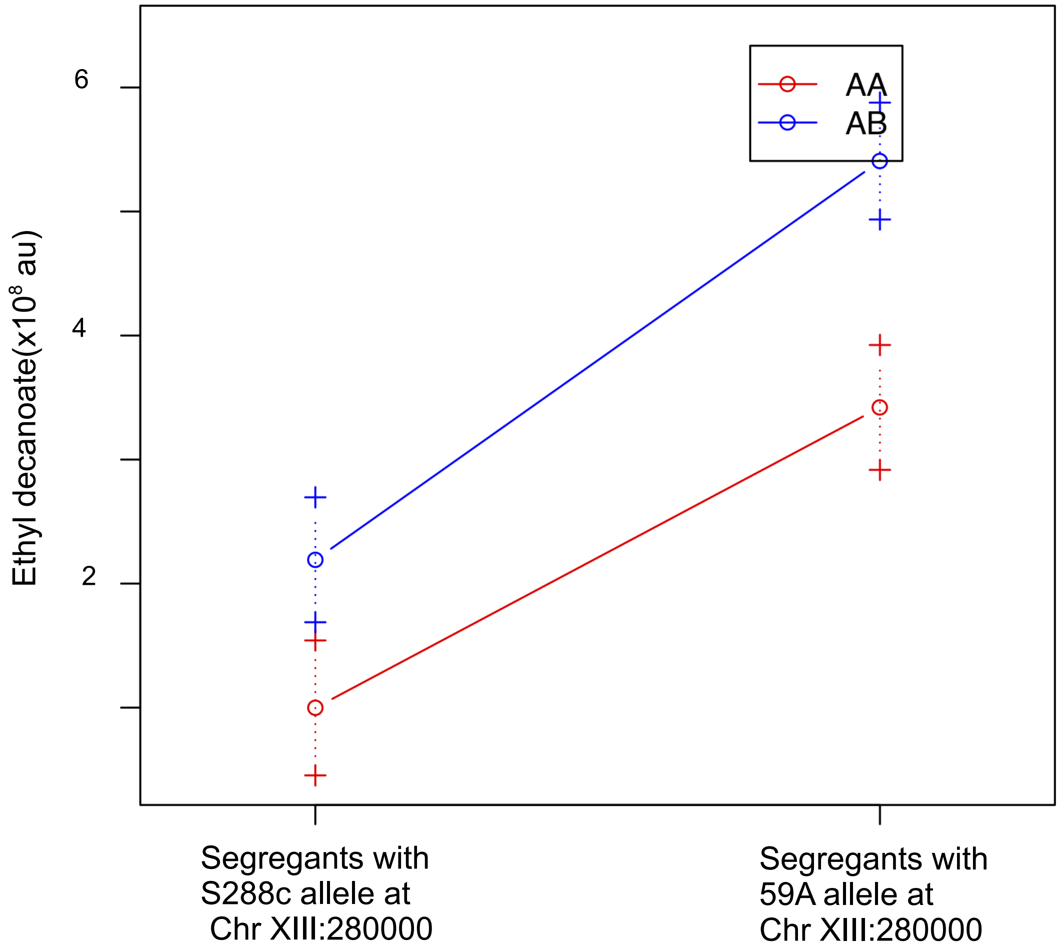

Results of QTL analysis with Haley-Knott regression for QTL (Normal model, 30 segregants)

		Mean ($x10^8$au)	SE	P value
ABZ1 (Chr VII:351000)		1.56	0.53	0.00728
marker at Chr XIII:280000		2.89	0.54	0.00001

Experiment B : Impact of flocculation on aroma production (additive model with interaction)

1- Effect of flocculation and locus Chr VII :351000 on dodecanoic production by segregants containing S288C or 59A allele (non flocculant strains: 0 in red, flocculant strains : 1 in blue)

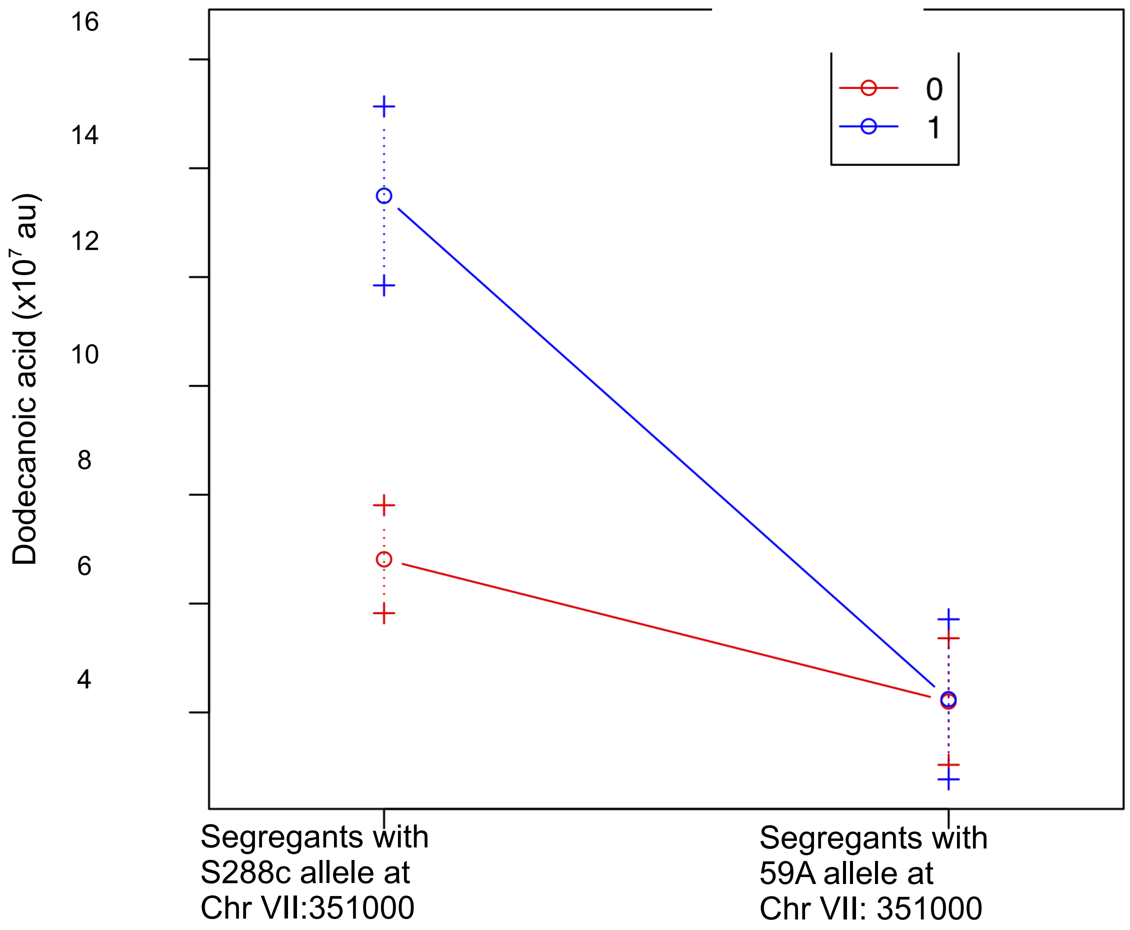

Results of QTL analysis with Haley-Knott regression for QTL (Normal model, 30 segregants)

		Mean ($x10^7$au)	SE	P value
Flocculation		3.77	11.5	0.00125
marker at Chr VII : 351000		-4.32	17.39	0.00002
interaction		-7.07	27.48	0.01666

Experiment B : Impact of the polymorphism of *ABZ1* on aroma production (additive model)

1- Effect of *ABZ1* locus and locus Chr XIII:280000 on ethyl-octanoate production by segregants containing S288C or 59A allele (S288C-ABZ1allele : AA in red, 59A-ABZ1allele: AB in blue)

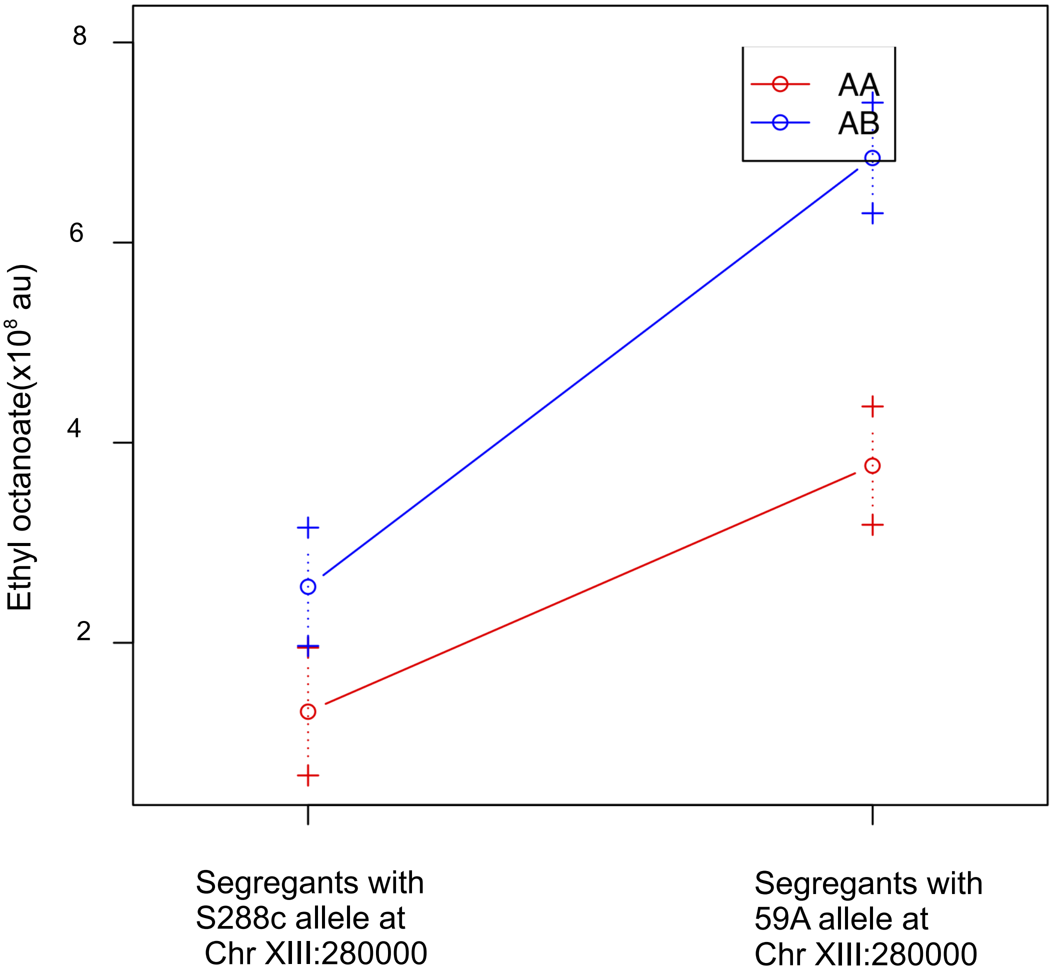

Results of QTL analysis with Haley-Knott regression for QTL (Normal model, 30 segregants)

		Mean (x10^{8}au)	SE	P value
ABZ1 (Chr VII:351000)		2.16	0.62	0.00198
marker at Chr XIII:280000		3.50	0.63	0.00001

2- Effect of *ABZ1* locus and locus Chr XIII:280000 on ethyl-decanoate production by segregants containing S288C or 59A allele (S288C-ABZ1allele : AA in red, 59A-ABZ1allele: AB in blue)

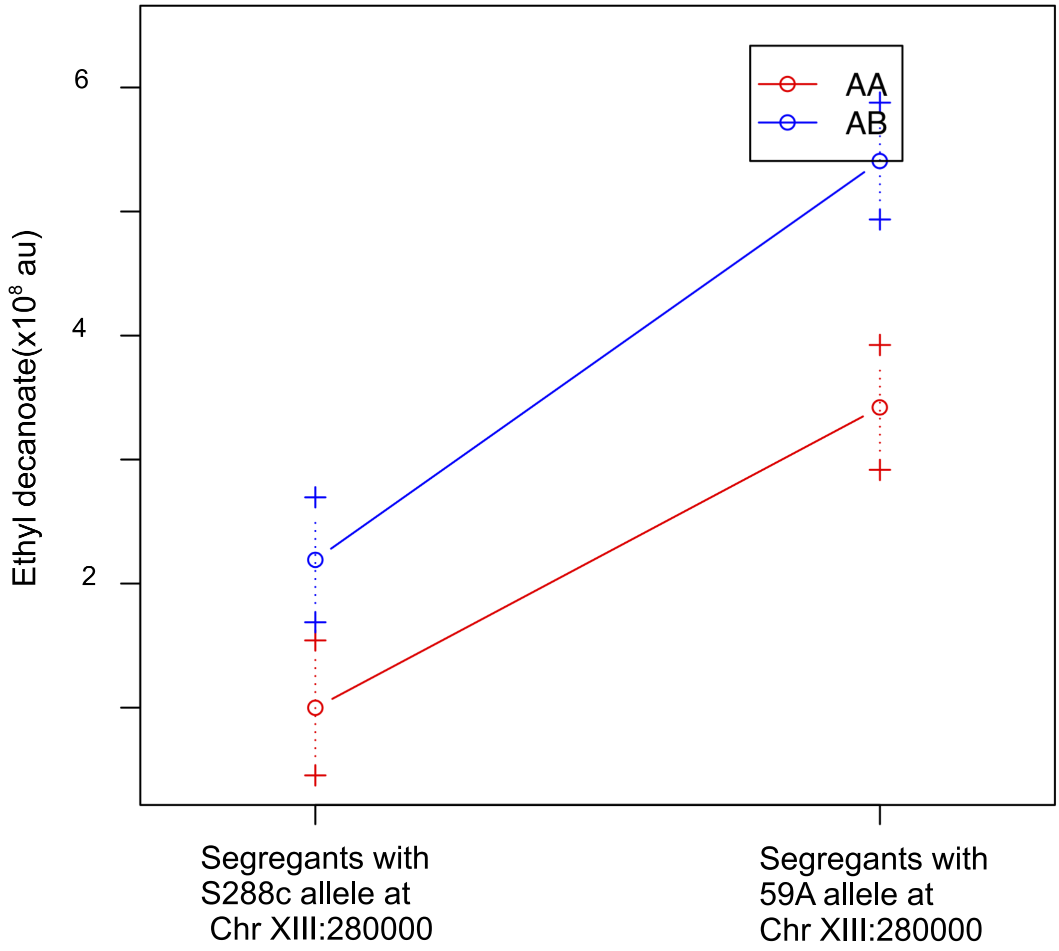

Results of QTL analysis with Haley-Knott regression for QTL (Normal model, 30 segregants)

		Mean ($x10^8$au)	SE	P value
ABZ1 (Chr VII:351000)		1.56	0.53	0.00728
marker at Chr XIII:280000		2.89	0.54	0.00001

Variability of fermentation length and flocculation ability of the different segregants

	Flocculation	Fermentation length in experiment A (hours)
**Parental strains**		
59A	0	351
S288C	0	811
**Segregants**		
10b	1	534
14a	1	783
14b	0	627
16a	0	797
16b	1	691
16c	0	641
16d	0	627
17a	1	534
18b	0	351
18c	0	441
19b	0	470
19c	0	627
1b	0	452
20a	0	351
20b	1	431
22a	0	627
22c	0	470
22d	1	351
23b	1	617
24a	0	617
25a	0	783
28b	0	351
29c	1	470
31b	0	627
3a	1	351
4c	1	431
5a	0	525
6b	0	351
7a	0	410
9a	0	470
