# Supplementary material for: QTL mapping of the production of wine aroma compounds by yeast
Source: BMC Genomics. 2012 Oct 30;13:573. doi: 10.1186/1471-2164-13-573 (PMC3575298; doi:10.1186/1471-2164-13-573)
Supplement: Additional file 3: Table S3 — Effect of the addition of p-aminobenzoic acid on the production of 2phenylethanol and 2-phenylethanolacetate. [file 1471-2164-13-573-S3.pdf]

	2- Phenyl ethanol (microg/l)	2-Phenyl ethanol acetate (microg/l)
**Control MS300**		
59A-ABZ1	214.7 ± 24.1	118.3 ± 22.3
S288C-ABZ1	93.6 ± 5.8	33.7 ± 2.4
pvalue	0.001	0.003
**MS300+pAMB(1mg/l)**		
59A-ABZ1	253.22 ± 25.2	180.4 ± 38.7
S288C-ABZ1	149.1 ± 39.3	183.8 ± 28.6
pvalue	0.018	0.910
